# Synergy of epidermal growth factor (EGFR) and angiotensin II (AT1R) receptor determines composition and temporal pattern of transcriptome variation

**DOI:** 10.1007/s00018-021-04065-5

**Published:** 2021-12-18

**Authors:** Barbara Schreier, Virginie Dubourg, Stefanie Hübschmann, Sindy Rabe, Sigrid Mildenberger, Michael Gekle

**Affiliations:** Julius-Bernstein-Institute of Physiology, Martin Luther University Halle-Wittenberg, Magdeburger Strasse 6, 06112 Halle (Saale), Germany

**Keywords:** Epidermal growth factor receptor, Angiotensin II receptor type 1, Serum response factor, Myocardin-related transcription factor, Signaling synergy, Vascular biology

## Abstract

The tyrosine kinase receptor EGFR and the G-protein-coupled receptor AT1R induce essential cellular responses, in part via receptor crosstalk with an unknown role in nuclear information transfer and transcription regulation. We investigated whether this crosstalk results in linear, EGFR-mediated nuclear signalling or in parallel, synergistic information transfer leading to qualitative and temporal variations, relevant for gene expression and environment interaction. AT1R and EGFR synergistically activate SRF via the ERK1/2-TCF and actin-MRTF pathways. Synergism, comprised of switch-like and graded single cell response, converges on the transcription factors AP1 and EGR, resulting in synergistic transcriptome alterations, in qualitative (over-additive number of genes), quantitative (over-additive expression changes of individual genes) and temporal (more late onset and prolonged expressed genes) terms. Gene ontology and IPA^®^ pathway analysis indicate prolonged cell stress (e.g. hypoxia-like) and dysregulated vascular biology. Synergism occurs during separate but simultaneous activation of both receptors and during AT1R-induced EGFR transactivation. EGFR and AT1R synergistically regulate gene expression in qualitative, quantitative and temporal terms with (patho)physiological relevance, extending the importance of EGFR-AT1R crosstalk beyond cytoplasmic signalling.

**Supplementary Information:**

The online version contains supplementary material available at 10.1007/s00018-021-04065-5.

## Introduction

The epidermal growth factor receptor (EGFR) family consists of four related tyrosine kinase receptors EGFR (ErbB1), ErbB2, ErbB3 and ErbB4 [1] that form homo- and heterodimers. EGFR controls various signalling modules and their downstream targets, thereby affecting transcriptional regulation and finally, e.g. cell proliferation, survival, differentiation, migration and matrix homeostasis [2].

In addition to its classical ligands, EGFR is also subject to activation by crosstalk with other receptors—a mechanism called transactivation [3]. Both mechanisms may have pathophysiological consequences that include cell proliferation and parainflammatory dysregulation of tissue homeostasis, leading for example to vascular dysfunction and remodelling or renal tubulointerstitial alterations. EGFR transactivation is supposed to be responsible for angiotensin II (AII) induced pathophysiological effects in the reno-cardiovascular system. These are mediated predominantly by the G-protein AT1 receptor (AT1R) that couples to Gq or G12/13 [4] and involves EGFR transactivation via A Disintegrin And Metalloproteinase (ADAM) metalloproteinase domain 17 or cSrc kinase [3, 5]. In the case of ADAM, shedding and binding of Heparin-binding EGF-like growth factor (HB-EGF) activates EGFR whereas cSrc leads to direct EGFR-phosphorylation [2]. Recently an additional mechanism of AT1R-EGFR interaction, AT1R-EGFR heteromerization leading to recruitment of Grb2, has been proposed [6]. Besides, AT1R-EGFR synergy independent of transactivation or receptor interaction has been described [7–10] without comprehensive characterisation of the postreceptor steps involved.

Pharmacological EGFR inhibition and conditional EGFR knockout models [11–23] showed physiological and pathophysiological relevance of EGFR transactivation for reno-cardiovascular AT1R signalling. As reviewed by Forrester et al. [4], a great variety of cell types co-express EGFR and AT1R. These include vascular cells, cardiac cells, renal cells, adipocytes, immune cells, cells of the central nervous system. Since virtually every cell type expresses EGFR endogenously, any cell that expresses AT1R co-expresses EGFR and AT1R and is subject to a potential synergy.

Although AT1R-EGFR interaction with respect to proximal signalling in cells of the reno-cardiovascular system is well studied, our understanding regarding the consequences of this interaction in terms of information transfer to the nucleus, transcription regulation and finally the transcriptome is still limited. It is not clear whether AT1R-EGFR transactivation leads to (i) a linear, EGFR-triggered, nuclear signalling or whether transactivation induces (ii) parallel AT1R and EGFR signalling leading to (iii) synergistic effects, as it would be also the case during separate but simultaneous activation by external ligands (EGF and AII). Understanding of these mechanisms is of importance because nuclear information transfer affects gene expression with major impact on cell fate.

In the present study, we investigated the potential interaction of EGFR and AT1R with respect to transcriptional regulation, underlying signalling pathways and the response mode (digital versus analogue) at the single cells level, with a special focus on the serum response factor (SRF), AP1 and EGR. SRF is a mammalian transcription factor that binds to a DNA *cis* element known as the CArG box. It responds to two distinct types of signalling cascades (GPCR-induced Rho signalling and growth factor-induced Ras/MAPK kinase signalling), mediates expression of distinct target genes [24] and regulates, for example, switches from a contractile to a proliferative phenotype of vascular smooth muscle cells [25–27]. Recently, a study in fibroblasts identified 960 serum-responsive SRF target genes [28]. The dual responsiveness, which is at least in part the result of two families of SRF cofactors (the ternary complex factors, TCFs and the myocardin-related transcription factors, MTRFs), regulated by the two signalling pathways, makes SRF an ideal point of intersection for combinatorial EGFR and AT1R interaction. Furthermore, we assessed the consequences for known SRE-regulated genes and functions as well as for the transcriptome by RNA-seq.

## Results

### EGFR-induced SRF activation

Under control conditions, SRE activity was stable for at least 48 h (SF02 A, H; SF03 A, H). Addition of EGF led to a time- and concentration-dependent increase of the fraction of SRE-positive cells as well as of the activity of individual positive cells (SF02 A, B; SF03 A, B). In depth analysis of the EGF action revealed that its effect resulted mainly (2/3) from a switch-like (digital) activation (i.e. activating otherwise inactive cells) and to a minor extend (1/3) from a graded (analogue) response (i.e. enhancing the activity of already activated cells) in all cell types. With respect to the concentration dependence, we observed no major differences for digital and analogue activation. By contrast, the digital response was faster as compared to the analogue response (SF02 A, SF03 A). We obtained a similar time course pattern for the PKC activator PMA, a classical stimulator of SRF activity (SF02 H, SF03 H).

EGFR has been shown to stimulate SRE-dependent transcription via the MEK/ERK1/2 and via the latrunculin B sensitive MRTF pathway [25]. Inhibition of the EGFR kinase (AG1478) and the MEK1/2 kinases (U0126), as shown in SF02 C and D and SF03 C and D prevented EGF-induced SRE activation. Furthermore, latrunculin B led to a significant reduction in EGF-induced SRE activation (SF02 E, SF03 E). Inhibition of Rho-associated kinase (ROCK) by Y27632 or protein kinase C (PKC) by BIM exerted no effect on EGF-induced SRE activation (SF02 F, G and SF03 F, G). The cytoskeleton interfering drug cytochalasin D exerted no effect either (data not shown).

For comparison, we tested the effect of PMA (SF02 H-N and SF03 H-N). PMA induced a fast and strong SRF activation. In depth analyses of the PMA action revealed that the effect resulted mainly from a switch-like (digital) activation and to a minor extend from a graded (analogue) response. PMA-induced SRF activation was sensitive to inhibition of MEK1/2 kinases, PKC, ROCK and AG1478. Latrunculin B showed no reduction of the PMA effect (SF02 J-N and SF03 J-N).

### AT1R-induced SRF activation

**Fig. 1:**
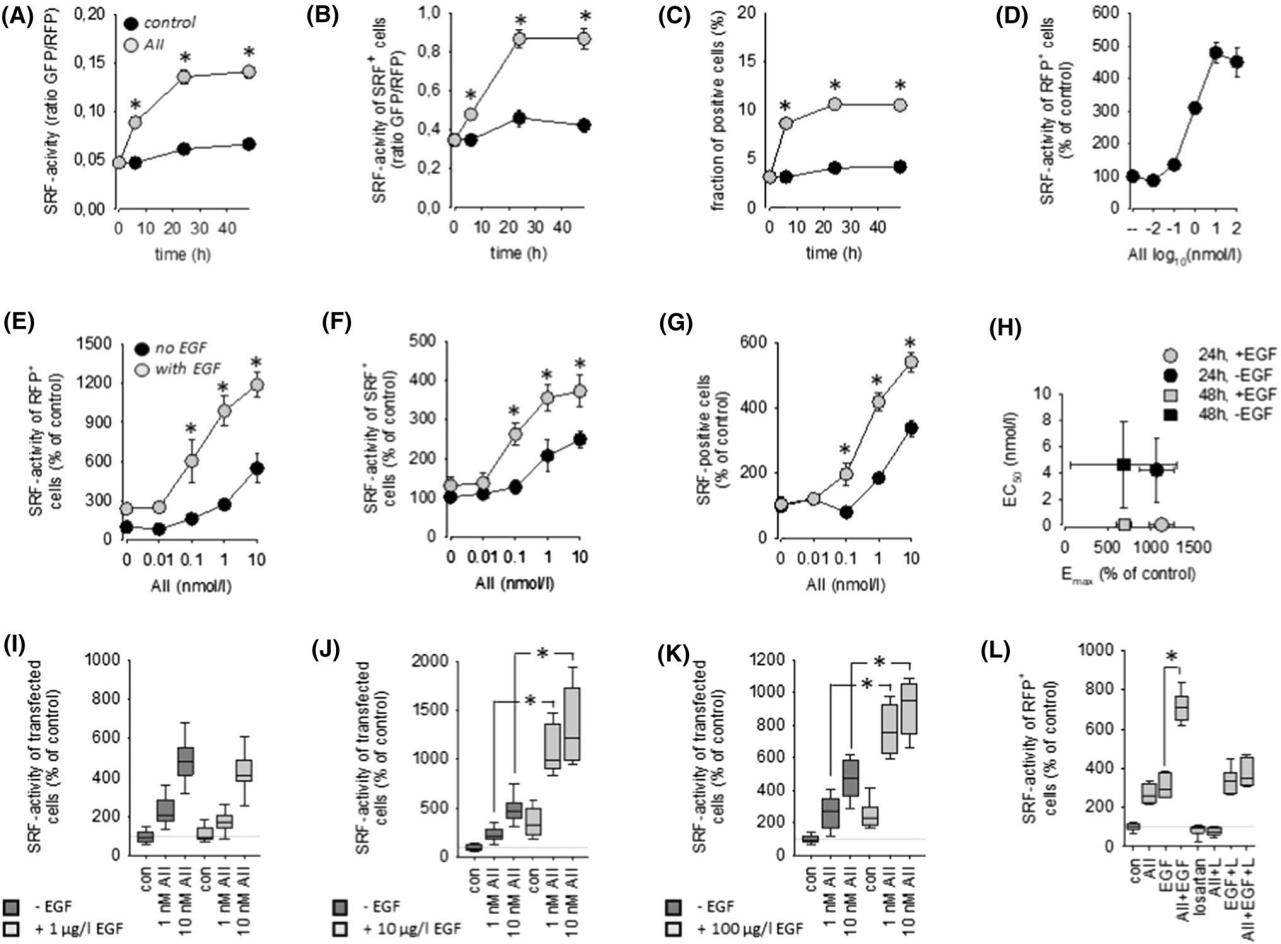
**(A)** Time-course of SRF activation by angiotensin II (1 nmol/l AII) in HEK293 cells transfected with AT1R. *N* = 24. **(B)** AII-induced SRF activity in responsive cells (= analogue response). **(C)** AII-induced percentage SRF-positive cells of transfected cells (= digital response). **(D)** Concentration–response curve of SRF activation by AII after 24 h. *N* = 18. **(E)** Synergistic action of 10 µg/l EGF and AII on SRF activity (*N* = 18; *t* = 24 h). **(F)** Synergistic SRF activation in responsive cells (= analogue response). **(G)** Synergistic increase of the percentage of SRF-positive cells (= digital response). **(H)** Estimation of the maximum effect (Emax) elicited by AII in the absence and presence of EGF and of the half-maximum AII concentration (EC50) from the data in panel **(E)** and SF05 (mean ± SD). **(I)** AII-induced SRF activity in the absence and presence of 1 µg/l EGF (*N* = 18; *t* = 24 h). **(J)** AII-induced SRF activity in the absence and presence of 10 µg/l EGF (*N* = 18; *t* = 24 h). **(K)** AII-induced SRF activity in the absence and presence of 100 µg/l EGF (*N* = 18; *t* = 24 h). **(L)** The action of 1 nmol/l AII and the synergistic action of 1 nmol/l AII + 10 µg/l EGF are prevented by the AT1R blocker losartan (**L**, 1 µmol/l; *N* = 12, *t* = 24 h). *p < 0.05 versus control, if not indicated otherwise

As expected from the literature, AII exerted no effect on SRE activity in HEK293 and HK2 cells not transfected with AT1R (SF01 E, F). AT1R transfection rendered cells sensitive to AII in a time- and concentration-dependent manner with respect to SRF activation (Figs. 1A–D and SF06 A, B). Transfection of AT1R per se had no effect (SF01 C). In depth analyses revealed that the AII effect resulted from a switch-like (digital) activation and to a similar relative extend from a graded (analogue) response in both cell types.

AII-induced SRF activation was reduced by U0126, BIM and latrunculin B but not by AG1478 or Y27632 (SF04 A-E and SF06 C-G). The resistance to AG1478 results from the inability of transmembrane EGFR transactivation in HEK293 cells [29], most probably due to the low level of endogenous HB-EGF expression (SF08 A). Thus, we have a system in our hands that allows separate and independent stimulation of the AT1R and EGFR pathways and thereby the controlled investigation of their potential convergence on SRF activity.

### Synergistic action of EGFR and AT1R with respect to SRF activation

Simultaneous stimulation of EGFR and AT1R by the addition of EGF and AII induced an increase in SRF-reporter activity that exceeded the sum of the individual effects significantly and was AII concentration-dependent (Fig. 1E, SF05 A and SF06 H). Analysis of AII concentration-dependency revealed a shift of the concentration required for the half-maximum effect (EC50), i.e. EGF sensitises the cells for AT1R activation with respect to SRF activation (Fig. 1H and SF06 I, J). The effects resulted from synergisms in switch-like (digital) and graded (analogue) responses (Figs. 1F, G, SF05 A-C and SF07 A-C). Furthermore, the synergism is also EGF concentration-dependent, observed at concentrations above 1 µg/l EGF (Figs. 1I–K, SF05 D-F and SF07 D-F) and could be prevented by losartan (Fig. 1L; a clinically used AT1R antagonist).

### Transactivation of EGFR via HB-EGF with respect to SRF activation

**Fig. 2:**
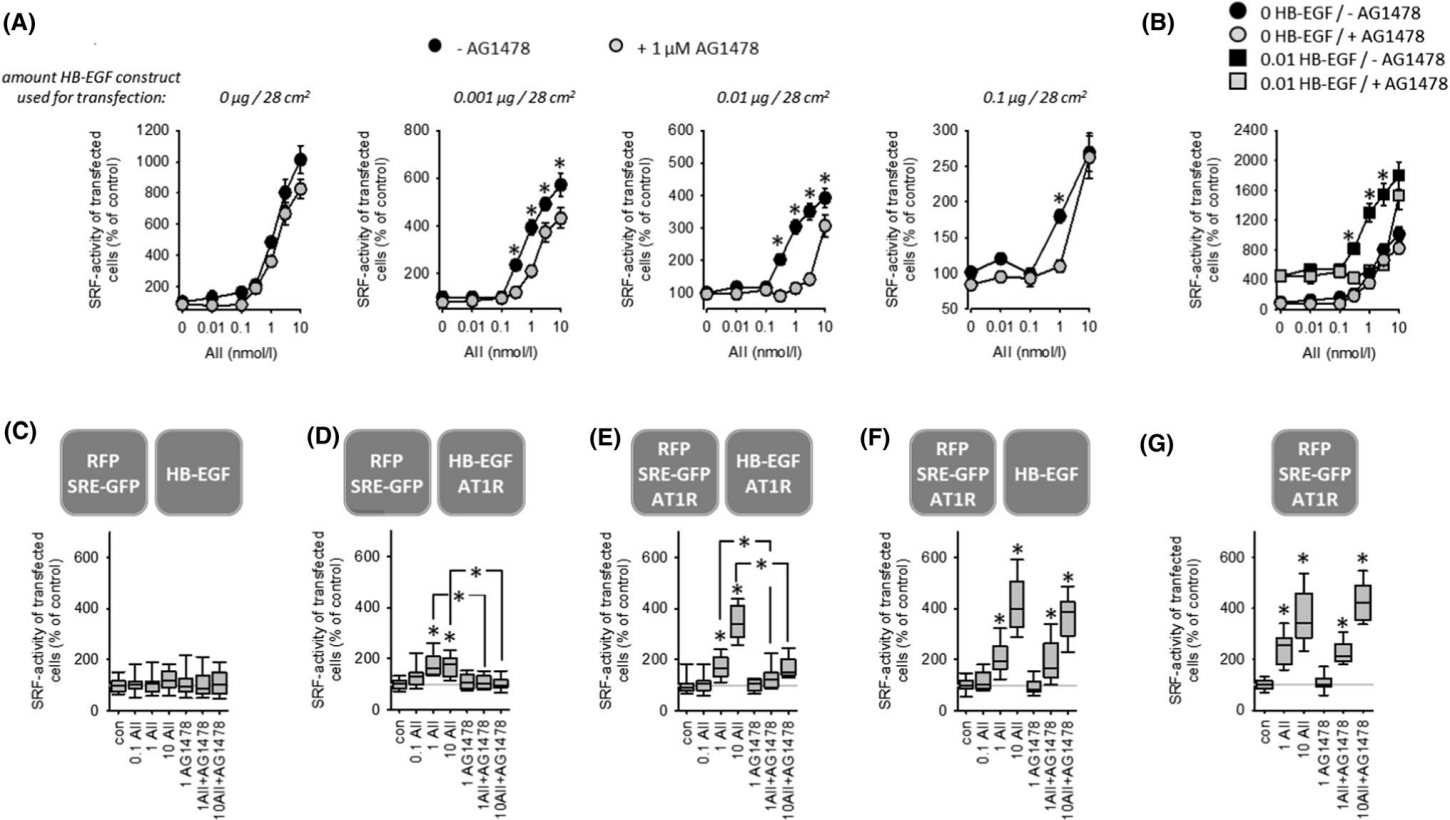
**(A)** In the absence of HB-EGF, AT1R-induced activation of SRF is independent of EGFR. HB-EGF expression leads to additional EGFR-mediated SRF activation by AT1R. *t* = 24 h. *N* = 18. *p < 0.05 versus “+AG1478”. **(B)** AT1R-induced activation of SRF normalised to control values in the absence of HB-EGF. HB-EGF expression leads to an increase in maximum SRF activation and increases the AII-sensitivity (left-shift of the dose–response curve). 1 µmol/l AG1478. *t* = 24 h, *N* = 18. *p < 0.05 versus “+AG1478”. **(C–F)** Coculture of reporter cells (transfected with SRE-GFP) with HB-EGF-transfected “donor “ cells shows that paracrine HB-EGF signalling also leads to a synergistic effect of AT1R and EGFR with respect to SRF activation. *t* = 24 h. **(C)** HB-EGF expressing donor cells without AT1R induce no AII-sensitivity in reporter cells. **(D)** Donor cells with HB-EGF and AT1R induce a moderate AII-sensitivity of reporter cells mediated by EGFR activation. **(E)** Additional expression of AT1R in reporter cells leads to synergistic (EGFR-dependent) SRF activation. **(F)** When AT1R is expressed in reporter but not in donor cells, the effect of AII is EGFR-independent. (**G**) This effect is similar to the reaction of reporter cells in monoculture. 1 µmol/l AG1478; 0.1, 1 or 10 nmol/l AII. *N* = 18. *p < 0.05 versus control, if not indicated otherwise

Transfection of HEK cells with HB-EGF, in addition to AT1R, enhanced the effect of AII and rendered its effect on SRF activity sensitive to EGFR inhibition (Figs. 2A, B, SF09 A, B). At high HB-EGF transfection doses, EGFR-dependency apparently decreased, due to the high control level of SRF activity during strong HB-EGF overexpression (SF08). There is a more than tenfold increase comparing 1 µg pHB-EGF with mock transfected cells. Figures 2B and SF09B show the approximately four to five fold increase of baseline SRF activity elicited by transfection with 0.01 µg HB-EGF. Thus, for example, 400% SRF activity in cells transfected with 0.01 µg pHB-EGF corresponds to 1600–2000% SRF activity in mock transfected cells (compare Fig. 2A, B). Analysis of the AII concentration-dependency (SF09 B) revealed a HB-EGF-induced increase of the maximum AII effect (Emax) and a shift of the AII concentration required for the half-maximum effect (EC50), i.e. HB-EGF sensitises the cells for AII with respect to SRF activation. These data indicate that AT1R-EGFR-synergism also works during EGFR transactivation but do not exclude the possibility of sole information transmission to SRF by EGFR. Therefore, we developed the experimental design of intercellular synergism.

### Intercellular synergism of EGFR and AT1R with respect to SRF activation

To confirm that EGFR-AT1R synergism also works during EGFR transactivation and paracrine signalling we performed experiments with a mix of two differentially transfected cell populations (Figs. 2C–G, SF10 A–E). One population was transfected with RFP and SRE-GFP (= reporter cells) and the second with HB-EGF (EGF donor cells). Exposure of this mixed cell population to AII exerted no effect on SRF activity (Figs. 2C, SF10 A). When EGF donor cells were additionally transfected with AT1R, AII led to an AG1478-sensitive SRF activation (Figs. 2D, SF10 B), due to HB-EGF shedding and paracrine signalling. Transfection of both cell populations with AT1R resulted in a significantly stronger AII-induced SRF activation (Figs. 2E, SF10 C), due to the synergism of AII and HB-EGF resulting from paracrine HB-EGF plus direct AII signalling. Figures 2 F, G and SF10D and E show that in the absence of AII-induced HB-EGF shedding or HB-EGF donor cells the effect of AII is AG1478 insensitive (= independent of EGFR). These results confirm that the two receptors synergize also during EGFR transactivation. Of note, the values of Fig. 2F cannot be compared directly with those in Fig. 2D, E, because the control level of SRF activity is significantly lower (approximately fivefold) under the conditions of Fig. 2E in comparison to Fig. 2D, E (Fig. SF10F). The different control values result most likely from intrinsic activity of transfected AT1R leading to a certain release of HB-EGF in the absence of AII.

### EGFR-AT1R synergism in A7r5 vascular smooth muscle cells

SF11 A–C shows that the synergism also occurs in vascular smooth muscle A7r5 cells, known to express low levels of endogenous AT1R [30, 31]. Thus, the synergism is relevant for differentiated cells with endogenous receptor expression.

### Pharmacology of EGFR-AT1R synergism

**Fig. 3:**
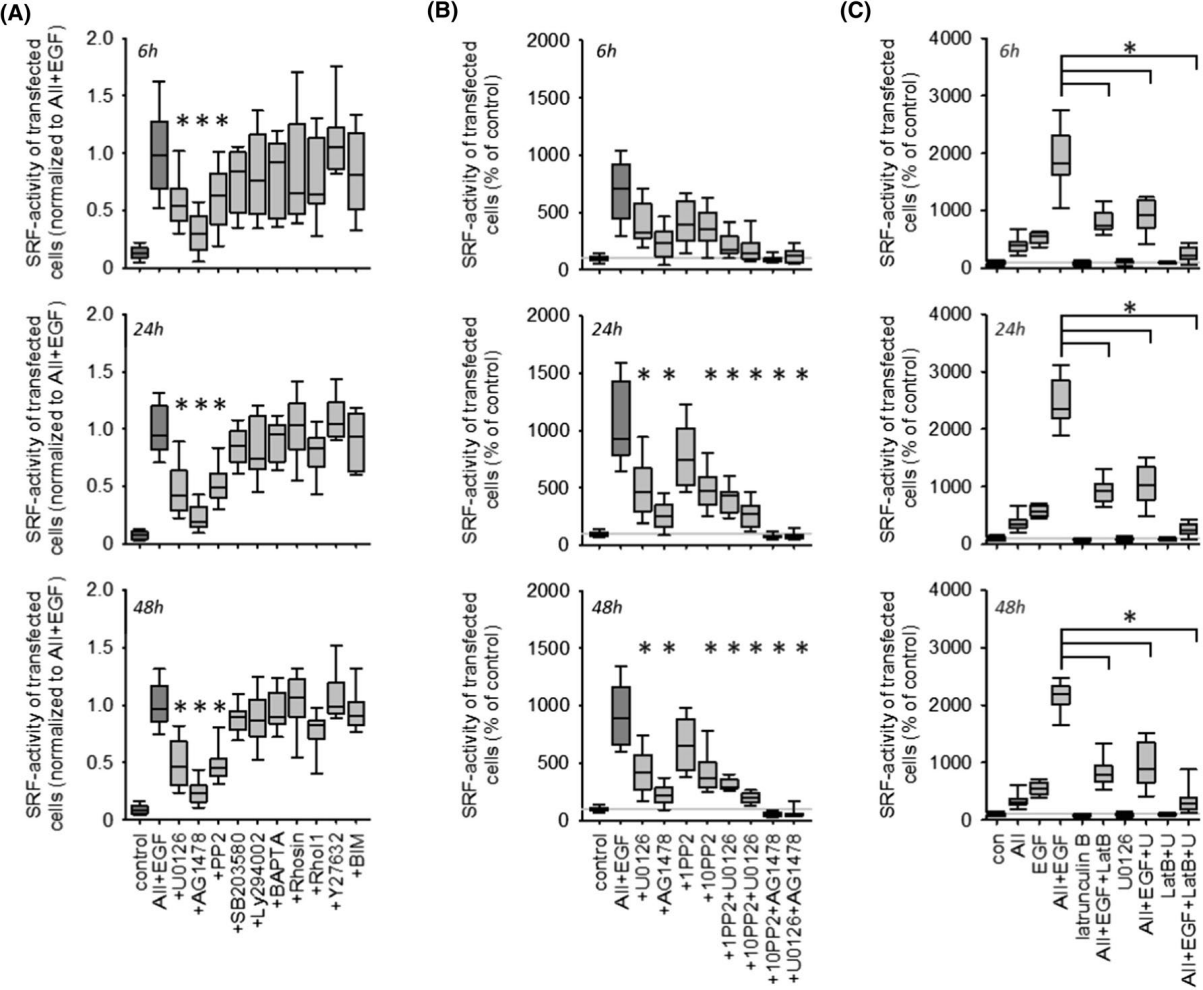
Pharmacology of ATR1-EGFR synergism with respect to SRF activation in HEK cells after 6, 24 or 48 h. **(A)** Pharmacological screening of putative involved pathway. Inhibition of EGFR kinase by 100 nmol/l AG1478, inhibition of ERK1/2-phosphorylation by 1 µmol/l U0126, inhibition of cSRC kinase family by 10 µmol/l PP2, inhibition of p38 kinase activity by 1 µmol/l SB203580, inhibition of PI3-kinase activity by 1 µmol/l Ly294002, Ca^2+^-chelation by 50 µM BAPTA-AM, Rho inhibition with 1 µmol/l Rhosin or 1 µg/ml Rho Inhibitor 1 (RhoI1, cell permeable C3 transferase), inhibition of Rho-kinase (ROCK) activity by 10 µmol/l Y27632 and inhibition of protein kinase C family by 100 nmol/l bisindolylmaleimide (BIM). *N* = 18. *p < 0.05 versus AII + EGF. **(B)** Additive inhibitory effects of 100 nmol/l AG1478, 1 µmol/l U0126 and 1 or 10 µmol/l PP2. *N* = 18. *p < 0.05 versus AII + EGF. **(C)** Inhibition of actin polymerization by 100 nmol/l latrunculin B (LatB) reduces the synergistic effect of EGF + AII substantially and act additively with U0126 to prevent SRF activation almost completely. *N* = 12

Figure 3A (and SF12 A) shows that inhibition of EGFR, MEK1/2 and cSRC kinases reduced the synergistic effect in HEK293 and HK2 cells significantly but not completely. Combined inhibition of MEK1/2 and cSRC exerted an additive inhibitory, but still not complete, effect (Figs. 3B and SF12 B). By contrast, combined inhibition of EGFR and MEK1/2 or cSRC prevented SRF activation completely.

Inhibition of actin-MRTF signalling by latrunculin B reduced synergistic EGF- and AII-induced SRF activation substantially but incompletely (Figs. 3C and SF12 C). Combined inhibition of actin-MRTF signalling and MEK1/2 abolished synergistic SRF activation almost completely (Figs. 3C and SF12 C). Interference with the actin cytoskeleton by cytochalasin D did not affect synergistic signalling (data not shown).

### EGFR- and ERK-phosphorylation

**Fig. 4:**
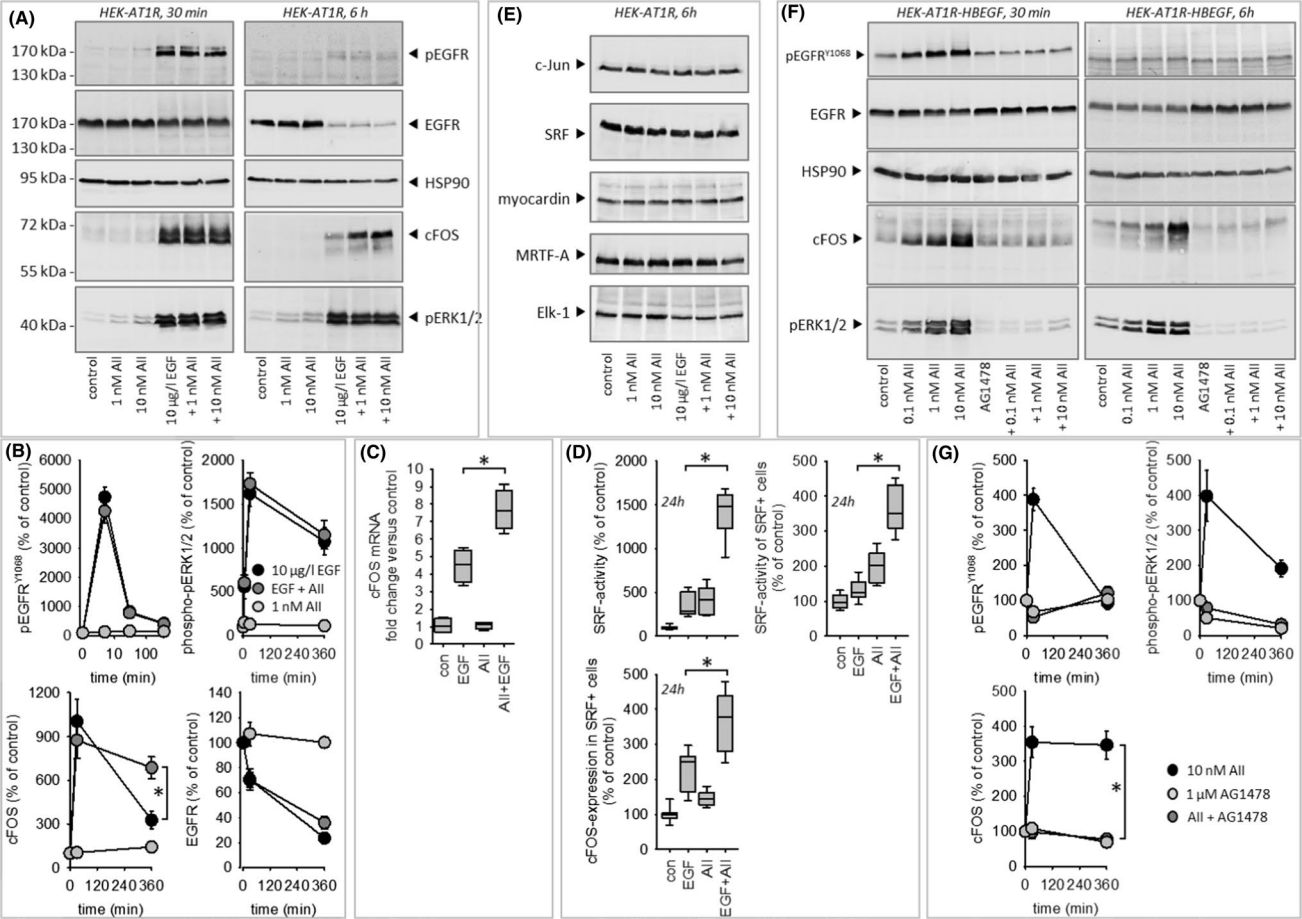
**(A–D)** Impact of EGFR- or AT1R activation on EGFR, phosphoEGFR, phosphoERK1/2, cFOS expression in HEK cells. **(A, B)** Results from immunoblot analysis of protein expression. **(C)** cFOS-mRNA expression determined by qRT-PCR. **(D)** SRF activity, determined by reporter gene assay and cFOS protein expression, determined by immunofluorescence. **(E)** Immunoblot analysis of c-JUN, SRF, myocardin, MRTF-A or ELK-1 expression showed no effect of EGF, AII or AII + EGF. Representative blots of *N* = 3. **(F, G)** Impact of EGFR transactivation by AT1R activation via HB-EGF on EGFR, phosphoEGFR, phosphoERK1/2 and cFOS protein expression in HEK cells. *p < 0.05. *N* for **(B, C, G)** = 4. *N* for **D** = 12

Figure 4A, B shows EGF-induced phosphorylation of pERK1/2 and EGFR, combined with a downregulation of absolute EGFR expression in HEK cells. AII exerted no synergistic effect regarding these three parameters. By contrast, we observed a synergistic effect with respect to cFos expression after 6 h at the protein (Fig. 4A, B) and at the mRNA level (Fig. 4C). Expression of cJun (forming the AP1-dimer with cFos) was not affected (Fig. 4E). Furthermore, EGF, AII or their combination (Fig. 4E) did not affect expression of the transcription regulating proteins SRF, myocardin, MRTF-A and Elk-1 in transiently transfected HEK293 cells.

When cells were transfected with HB-EGF in addition to AT1R, AII induced a strong ERK1/2-phosphorylation as well as EGFR-phosphorylation, prevented by AG1478. Furthermore, AII induced AG1478-sensitive cFOS expression in the presence of HB-EGF (Fig. 4F, G).

### cFos and SRF coincidence

Since the immunoblot data above do not allow conclusions regarding the cellular coincidence of SRF activation and cFOS induction, we tested the hypothesis that cells responding with SRF activation also show enhanced cFOS expression by fluorescence microscopy. Under control conditions, we observed cFOS staining in > 97% of the cells, indicative of constitutive basal expression. Coexposure of the cells to EGF and AII for 6 h induced the expected synergistic effect on SRF activity (Fig. 4D) and exerted a synergistic effect on cFOS expression in SRF-positive cells. This was not the case for SRF-negative cells.

### EGFR trafficking

Under control conditions, the membrane fraction of EGFR stayed virtually constant over the observation period of 55 min (SF13 A, D). In the presence of EGF, the membrane fraction decreased significantly after 20–25 min due to receptor internalisation. Appearance of EGFR in the perinuclear (cytoplasmic) region in a spot-like pattern (endocytic vesicles and early endosomes) and an increase in the perinuclear/cell EGFR ratio (SF13 B, E) accompanied this internalisation. The nuclear/cell EGFR ratio was not affected (SF13 F). AII application exerted no effect on the EGFR distribution per se but delayed EGF-induced EGFR internalisation and perinuclear appearance significantly (SF13 A, B, D, E), indicating a transient stabilising effect of activated AT1R for membrane EGFR that may lead to prolonged signalling. As shown in Fig. 4A, B, stimulation-induced degradation after 6 h was not affected by AII.

### EGFR-AT1R protein–protein interaction

Applying proximity labelling mediated by engineered ascorbic acid peroxidase (APEX) [32, 33], we analysed a potential protein–protein interaction of endogenous EGFR with transiently transfected AT1R-APEX after 60 min of stimulation. EGF reduced the EGFR-AT1R-interaction (SF13 G, H), most probably owing to EGFR internalisation. AII prevented this effect (SF13 G, H) in line with its retarding action on EGFR internalisation (SF13 A, D). In cells transfected with pcDNA3-APEX2-NES (non-specific interaction control) EGF, AII or EGF + AII exerted no effect.

### Transcriptome analysis

To investigate the impact of EGFR-AT1R-synergism on gene expression comprehensively and in detail, we generated HEK293 cell clones stably expressing AT1R. Two clones were responsive to AII with respect to SRF activity and ERK1/2-phosphorylation, confirming successful and functional AT1R expression (SF14 A–D). Furthermore, we observed synergism of AT1R and EGFR with respect to SRF activity but not to ERK1/2- or EGFR^Y1068^ phosphorylation in both clones (SF14 B–D). Thus, the clones confirm the results obtained with transiently transfected cells. For the subsequent investigation of gene expression, we used clone B6 that showed stronger AII responsiveness. Transcriptome changes were determined after 6 and 24 h incubation (control, 1 nmol/l AII, 10 µg/l EGF, AII + EGF, N = 5 for each condition).

**Fig. 5:**
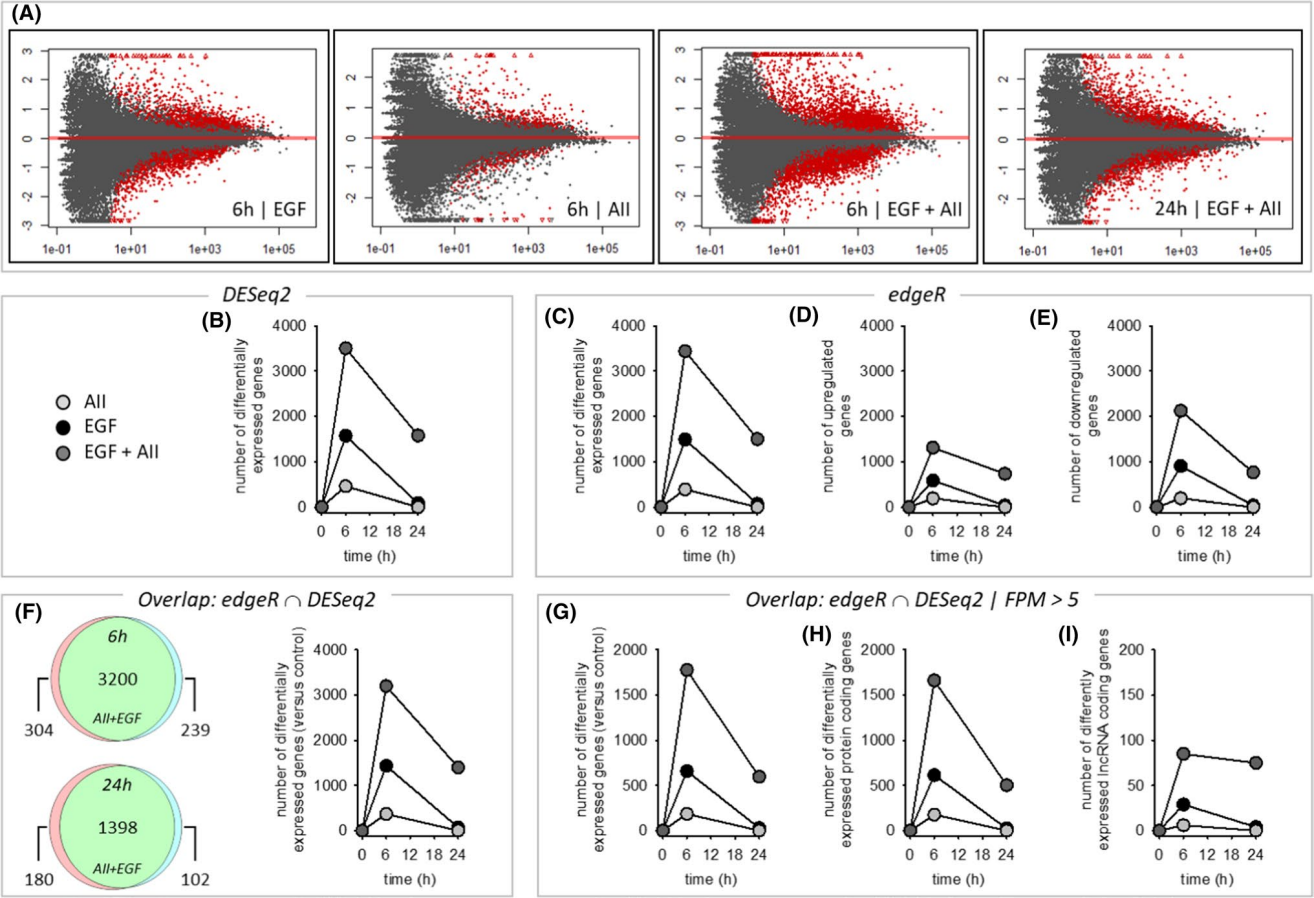

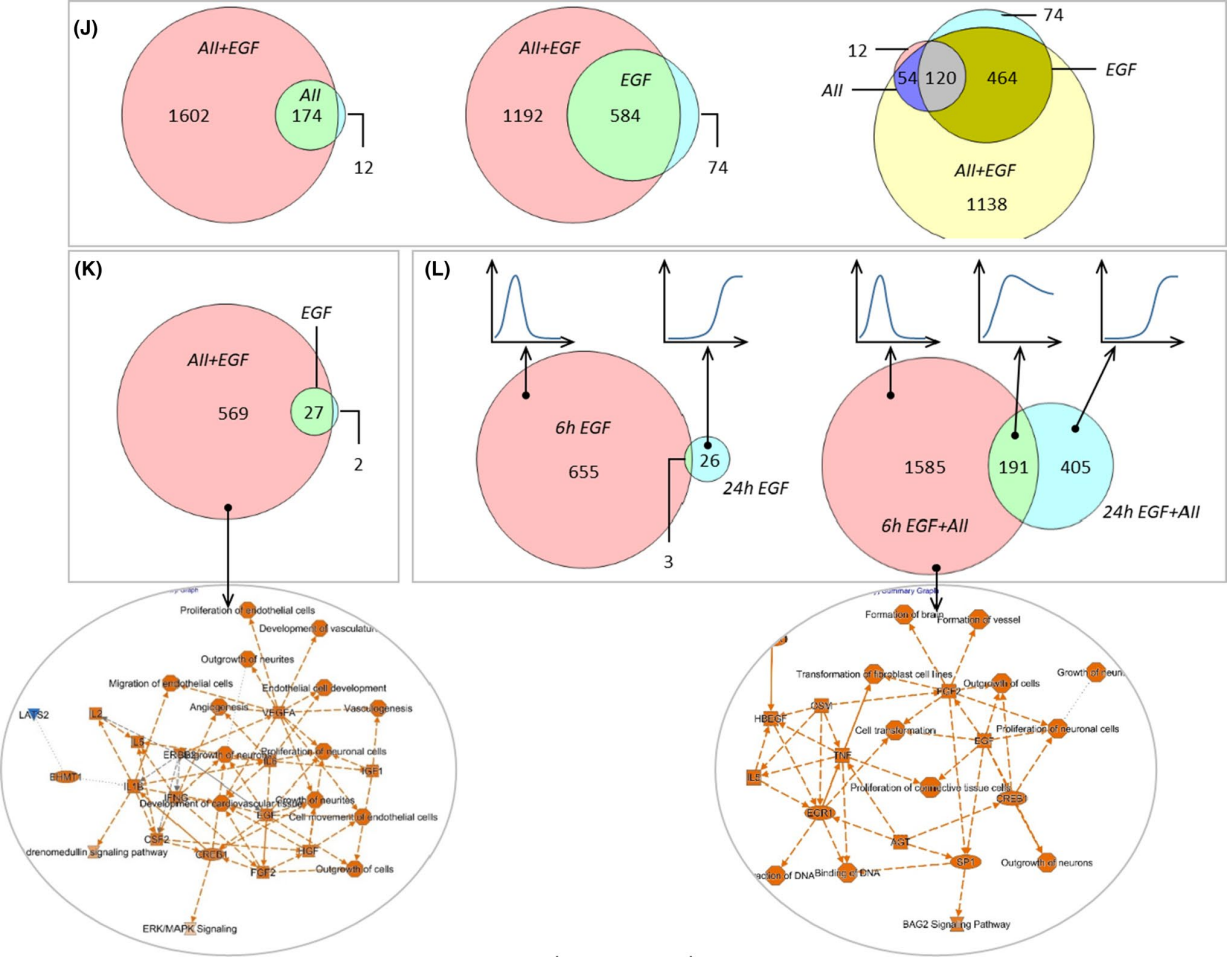

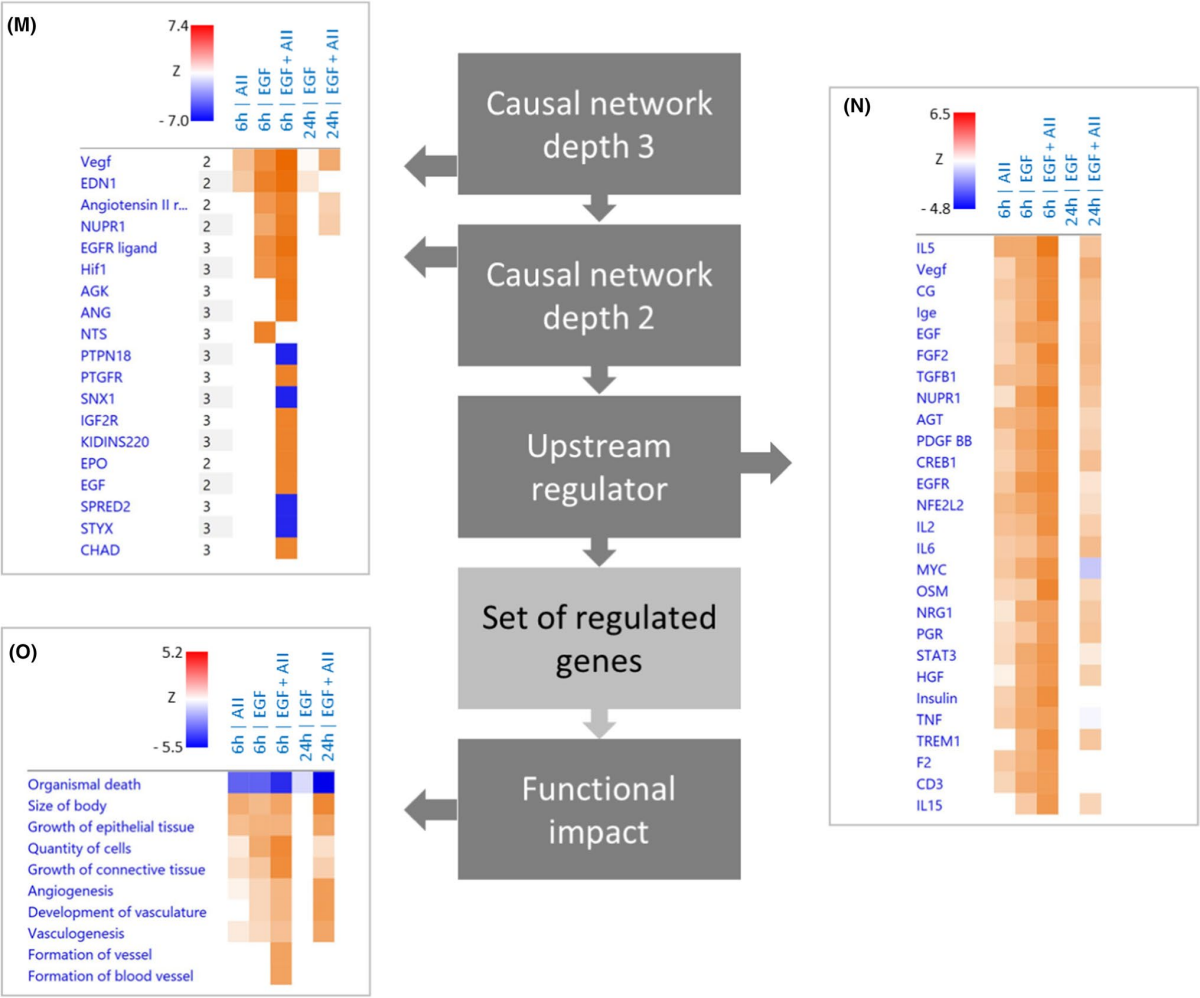
Effect of EGF (10 µg/l), AII (1 nmol/l) or AII + EGF on the transcriptome in HEK-AT1R-B6 cells, stably transfected with AT1R. Incubation periods = 6 h and 24 h. *N* = 5 for each condition. **(A)** Number of up- or downregulated RNAs (red dots) applying the mentioned thresholds (*y*-axis = log2FC; *x*-axis = FDR). **(B)** Number of differentially expressed genes according to DESeq2. **(C–E)** Number of differentially expressed, upregulated or downregulated genes according to edgeR. **(F)** Number of differentially expressed genes according to the intersection of DESeq2 and edgeR. **(G)** Number of differently expressed genes with an abundance FPM > 5 according to the intersection analysis. **(H)** Number of affected protein coding genes (FPM > 5). **(I)** Number of lncRNA coding genes (FPM > 5). **(J, K)** Comparison of gene expression regulation by 10 µg/l EGF or 1 nmol/l with regulation by simultaneous AII + EGF incubation after 6 h **(J)** and 24 h **(K)** in HEK-AT1R-B6 cells, stably transfected with AT1R. **(L)** Comparison of gene expression regulation after 6 h and 24 h by 10 µg/l EGF or 1 nmol/l AII + EGF in HEK-AT1R-B6 cells, stably transfected with AT1R. *N* = 5 for each condition. The circled graphs represent graphical summaries of the IPA^®^ analysis. For further details, see supplementary table ST01-ST05. **(M–O)** Comparative analysis by IPA^®^ of the five scenarios with differentially expressed genes. The B–H threshold was set to 0.01. **(M)** Causal networks of depth 2 or 3. |*z*-score| threshold was set to > 6. **(N)** Upstream regulator. |*z*-score| threshold was set to > 4. **(O)** Disease and biofunction subset ‘Physiological system development and function’. |*z*-score| threshold was set to > 3. For a full list of the analysis results, see supplementary table ST06

Figures 5A, B and SF15 A show the quantitative effects of AII, EGF and AII + EGF obtained by DESeq2 analysis, with a prominent synergistic action of AT1R and EGFR with respect to the number of regulated genes but also with respect to the SRF-responsive genes ARC, EGR1, EGR2, EGR3 and cFOS (SF15 B). Figure 5C–E shows the results obtained by EdgeR analysis, including a differentiation for up- and downregulated genes, and confirm the synergistic action. DESeq2 and EdgeR results overlap substantially (Fig. 5F) and we used the intersection for further analysis, applying an abundance threshold of 5 FPM to reduce transcription noise. More than 90% of differentially expressed genes were protein coding and less than 10% lncRNA (Fig. 5G–I). In summary, the data show that stimulation of AT1R and EGFR resulted in a synergistic action on the number of differentially expressed genes after 6 and after 24 h (SF15 D). In relative terms, the synergistic effect is especially prominent after 24 h, because AT1R activation alone was without effect and EGFR activation affected only a small number of genes. This effect represents an AT1R-induced prolongation of EGFR action on gene transcription. Synergism affects the number of genes and the time course of expression regulation (qualitative and temporal effect)

According to the qualitative analysis (Figs. 5J, K and SF16 A–C), the majority of genes affected by AT1R or EGFR after 6 or 24 h are also part of the portion of genes regulated by the synergistic action. The extent of expression regulation in these overlap groups was slightly higher during synergistic action (correlation slope 1.2–1.5). There was no alteration of the direction of regulation by the synergism. Thus, synergism results in the recruitment of additional genes for regulation and in enhanced regulation of genes already regulated by AT1R or EGFR alone.

The intersection of regulated gene sets after 6 and 24 h was ~ 10% (based on the 24 h value) during EGFR activation but ~ 30% during simultaneous activation of AT1R and EGFR (Figs. 5L, SF16 C). Thus, during sole EGFR activation, there are two almost distinct temporal groups of regulated genes, early but transient and late ones. Synergism not only increases the number of regulated early and late genes but also the portion of genes induced early and “permanently”. Thus, synergism adds a third group of genes with respect to the time course of regulation (Figs. 5L, SF16 C). The majority of genes showed monophasic up- or downregulation but there were also some genes with biphasic regulation.

Finally, we performed gene ontology (GO) enrichment and IPA^®^ pathway analysis (thresholds: adjusted *p* value ≤ 0.01 and enrichment ≥ 2 for GO enrichment; |*z*-score|≥ 2 and B–H-corrected *p *value ≤ 0.01 for IPA). After 6-h AT1R activation, 117 genes were up- and 69 downregulated. Whereas no major gene set enrichment was determined for downregulated genes, there was an enrichment of terms related to amino acid handling (metabolism and transport) and to EGR-mediated regulation of gene expression for the sets of upregulated and all genes (Supplementary tab. ST01). Pathway analysis (ST01) resulted in the enrichment of one canonical pathway, namely tRNA charging. Upstream regulator analysis identified four transcription regulators with a z-score > 2 (ATF4, XBP1, NFE2L2, TCF4). ATF4, XBP1, NFE2L2 are response factors to unfolded protein and oxidative stress and play important roles for ER homeostasis and proteostasis. Thus, these results reflect to a certain extend the hypertrophic action of AII in pathological situations.

After 6-h EGFR activation, 381 genes were up- and 277 downregulated (ST02). There was GO enrichment for downregulated genes of terms related to KLF3-mediated regulation of gene expression, however, with marginal adjusted p values. For upregulated genes as well as for the group of all regulated genes, there was a very strong enrichment of terms related to EGR-mediated regulation of gene expression. Pathway analysis (ST02) resulted in no canonical pathway with a z-score above the threshold. Upstream regulator analysis identified, besides the expected growth factors (EGFR is predicted with the lowest B–H-corrected *p* value), 13 transcription regulators with a z-score > 2 (including CREB, CTNNB1, EGR, EP300 & CREBBP, HIF1A, HIF2A (EPAS), MYC, STAT3). The activity of two transcriptional repressors is predicted to be reduced (CIC, KDM5B). Furthermore, 10 cytokines (TNF, IFNG, IL5, IL2, CSF2, IL4, IL1B, IL6, TNFSF11, CSF1) and two ligand-dependent nuclear receptors were predicted, reflecting the known participation of EGFR in cellular signalling of this molecule type. These results are in good agreement with known EGFR functions and underpin the validity of the analysis.

After 6-h simultaneous EGFR and AT1R activation, 907 genes were up- and 869 downregulated (ST03). The enrichment for downregulated genes of terms related to KLF3 was intensified. Regarding upregulated genes, the number of terms with significant enrichment increased substantially compared to single EGFR activation. Besides a more pronounced enrichment of terms related to EGR-mediated regulation of gene expression (B–H-corrected *p* value < 10^–42^), terms related to SRF-mediated regulation also showed significant enrichment. Furthermore, terms related to decreased oxygen levels and the VGEF pathway were enriched. Pathway analysis resulted in no canonical pathway with a *z*-score above the threshold. Upstream regulator analysis (ST03) identified 27 transcription regulators with a *z*-score > 2, with the lowest adjusted p values for HIF1 and HIF2A (EPAS), corresponding to the terms related to low oxygen levels. In addition to single EGFR activation, the following transcription regulators showed *z*-scores above 2 during simultaneous AT1R and EGFR activation: ATF4, CCND1, FLI1, GLI1, HNF4A, JUN, SP1 and SRF. In addition, three microRNAs were predicted to be inhibited with a *z*-score < -2 (miR-16-5p, let-7, miR-10).

In the group of genes regulated by 6-h simultaneous stimulation of AT1R and EGFR but not by sole stimulation of EGFR (Figs. 5J, SF16 A), 579 were up- and 613 downregulated (Supplementary Tab. ST04). There was an enrichment for downregulated genes especially of terms related to SP1- and SP3-mediated regulation of gene expression. For upregulated genes as well as for the group of all regulated genes, there was an enrichment of terms related to EGR-mediated regulation of gene expression. Furthermore, terms related to decreased oxygen levels and the VGEF pathway were enriched (ST04).

After 24-h simultaneous EGFR and AT1R activation, 363 genes were up- and 233 downregulated (Figs. 5K, L and SF16 B, C; ST05). There was only minor enrichment for downregulated genes of terms related to NRF1-mediated regulation of gene expression with marginal adjusted *p* values. For upregulated genes, there was a moderate enrichment of terms related to EGR-mediated regulation of gene expression. Pathway analysis resulted in no canonical pathway with a *z*-score above the threshold. Upstream regulator analysis identified six transcriptional regulators with a *z-score* > 2 (ATF4, JUN, SP1, CREB1, NUPR1, FOS). Furthermore, five cytokines (IFNG, IL5, IL2, CSF2, IL1B) were predicted as upstream regulators (ST05).

The comparative pathway analysis are shown in ST06 and Fig. 5M–O (only the top results with respect to *z*-score are shown; the complete set of results is given in ST06) and summarised for qualitative synergistic effects in supplementary figure SF17A–C. We identified several early (6 h) and late (24 h) additional terms (i.e. enriched only after simultaneous stimulation) for upstream regulation related to (hypoxic) cell stress response and fibrogenesis (Figs. 5M, N and SF17A-B). For “disease & function”, additional terms are related among others to vascular biology (Fig. 5O). Late-onset terms (SF17B) included additionally alterations of cellular carbohydrate uptake and possibly metabolism. Applying prolongation conditions (SF17C) resulted several growth factor related terms, indicating that AII leads to a temporal extension of EGFR effects.

### EGR-, FOS-, ARC- and TGFß-expression, and EGR- and AP1-activity

**Fig. 6:**
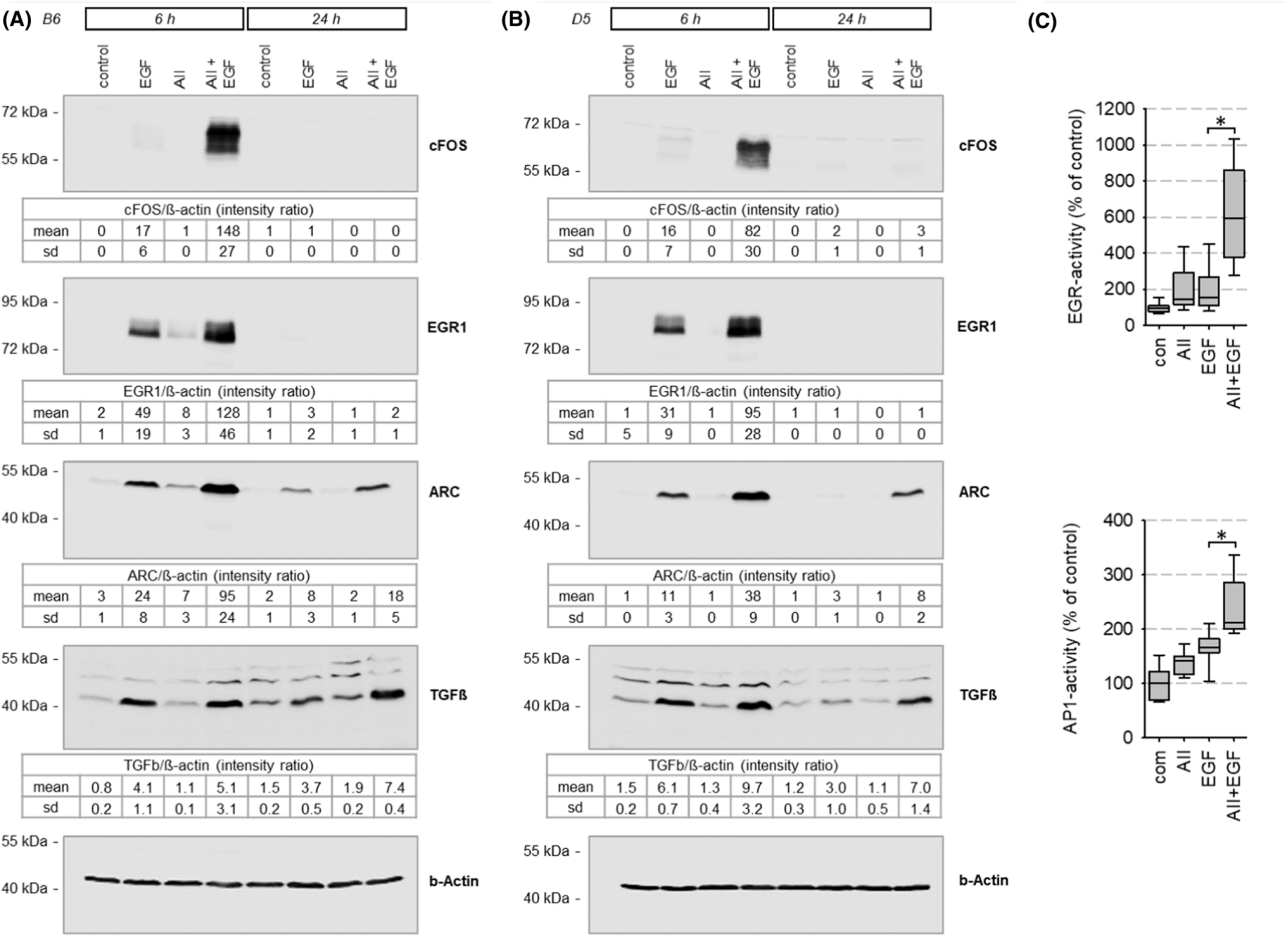

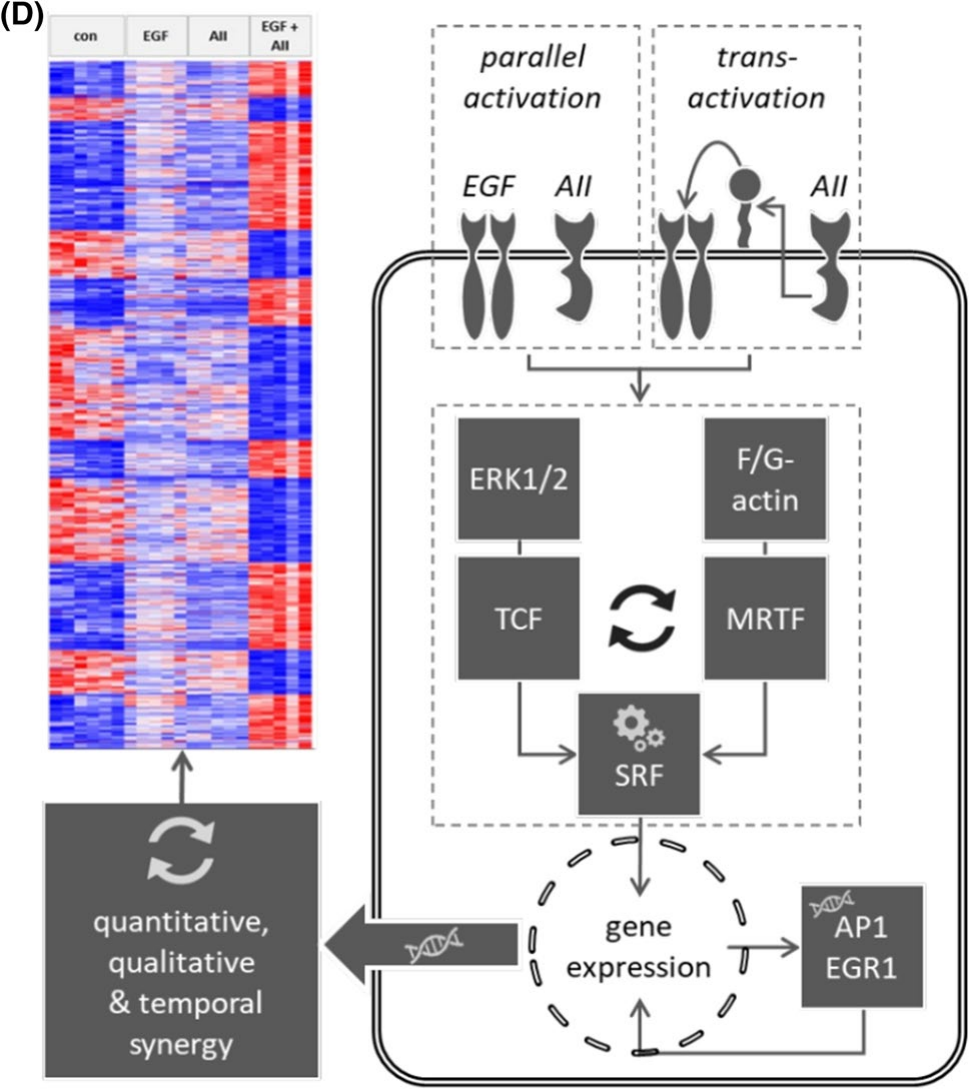
**(A, B)** Synergistic effect of EGFR and AT1R on protein expression of known SRF target genes in stable transfected HEK293-AT1R clones B6 and D5. The tables show the data from three independent experiments. Synergistic effect of EGFR and AT1R on **(A)** AP1- and **(B)** EGR-mediated transcriptional activity in HEK293 cells transfected with AT1R. T = 24 h. 1 nmol/l AII, 10 µg/l EGF, N(AP1) = 12, N(EGR) = 24. *p < 0.05. **(D)** Graphical summary of AT1R-EGFR-synergism

Figure 6A, B shows the synergistic effect of EGFR and AT1R activation with respect to cFOS, EGR1, ARC and TGFß protein expression in accordance with the effects on the mRNA level (ST03) in HEK293-AT1R clones B6 and D5. No synergistic effects were observed with respect Elk-1, phospho-Elk-1, myocardin, MRTF-A and B, nor with respect to intermediate-term downregulation of EGFR (SF18)

EGFR-AT1R synergism with respect to EGR1 and cFOS upregulation at the protein level also translated into enhanced EGR- or AP1-mediated transcriptional activity, determined by reporter assays (Figs. 6C, SF19). Since EGR and cFOS transcription factors are SRF-induced genes, these data underscore the evidence that the synergism observed at the level of SRF activity is of relevance for transcriptional regulation.

Finally, latrunculin + U0126 significantly reduced the synergistic upregulation of ARC expression (SF19).

## Discussion

Our study describes EGFR-AT1R synergy with respect to gene expression regulation by SRF, AP1 and EGR at the single cell level. Both receptors elicit an effect of their own, yet when activated simultaneously, the resulting effect is significantly larger than the sum of the individual effects. EGFR-AT1R synergy can occur independent of EGFR transactivation (simultaneous exogenous activation of both receptors) but also occurs during AT1R-induced EGFR transactivation (indirect simultaneous activation of both receptors) playing most probably a widespread role (Figs. 6D, SF20). Both of the two SRF activation pathways, actin-MRTF-SRF and the ERK1/2-TCF, mediate, at least in part, this synergy. Furthermore, our study shows at the single cell level that the extracellular signals EGF and AII, via EGFR and AT1R, are encoded heterogeneously in individual cells with respect to SRF activity, leading to two different categories of responses, i.e. switch-like (digital) or graded (analogue), similar to NFkB responses elicited by TNF [34]. The digital response appears somewhat faster and, in relative terms (fold change), somewhat stronger. Mere cell population data would not allow distinguishing these mechanisms, because digital and analogue responses appear similar at the population level, although the biological significance may vary substantially (compare 100/100 cells with a below threshold change to 10/100 with a tenfold stronger above threshold change that induced a transcriptional response). Digital responses allow dynamic encoding for groups of cells, whereas analogue responses enable single cells of dynamic encoding. The combination of cell individual analogue and digital responses allows more checkpoints, better fine-tuning and therefore enhanced transmission diversity and safety in cell ensembles. Furthermore, subpopulations of cells with different response pattern can exist, enhancing transmission versatility of cell assemblies further. From the viewpoint of a single cell, signal transmission works like a dimmer-switch. The switch is not simply turned “on” and “off”, but can be regulated to different intensities in the “on” state.

Mechanistic analysis of the synergistic effect at the single cell level, using SRF activity as readout, also revealed a combination of digital and analogue effects, i.e. a graded response with threshold [34] that requires EGFR and cSRC activity for initiation and involves actin-MRTF-SRF as well as ERK1/2-TCF signalling. Thus, the synergism works like a dimmer-switch, too. Our data on EGFR trafficking and EGFR-AT1R protein–protein interaction indicate that signalling interaction starts at the receptor level, although the mechanistic details will have to be investigated in future studies. However, different EGFR abundance due to decelerated degradation or enhanced EGFR-phosphorylation is not responsible. Simultaneous activation of EGFR and AT1R not only leads to synergistic activation of SRF and expression of selected SRF-regulated genes (e.g. cFOS, EGR, and ARC) but to the synergistic regulation of a substantial part of the transcriptome (> 1500 protein coding genes).

EGFR-AT1R synergism with respect to the transcriptome is apparent by the number of regulated genes that is much higher than the sum of genes regulated by EGFR or AT1R activation alone, i.e. synergism exerts a qualitative effect extending the group of regulated genes, with the repression of regulation for only a small number of genes. Furthermore, there is also a quantitative, although less pronounced, component because the extend of regulation for most of the genes affected by single as well as synergistic stimulation was larger when both receptors were activated, at least in the initial phase.

Besides influencing the identity of regulated genes, EGFR-AT1R synergism also affected the time course of transcription. During separate activation of AT1R or EGFR, the number of regulated genes decreased sharply, even to zero in the case of AT1R. In contrast, simultaneous stimulation of EGFR and AT1R resulted in 596 genes regulated after 24 h. In relative terms, this is a very strong synergistic effect because the number of regulated gene increases by a factor of ~ 20 (596 genes by EGF + AII versus 29 genes by EGF and 0 by AII). For 1/3 of the genes, synergism results in prolongation of regulation (i.e. regulated at 6 and 24 h; examples are ARC and TGFß) whereas for 2/3 of the genes synergism implied exclusive late onset regulation that was observed only for a very small number of genes during EGFR activation.

Gene ontology enrichment and IPA^®^ pathway analysis indicate that besides SRF, EGR and AP1 transcription factors are major targets of the synergistic action. The synergistic effects of EGFR and AT1R activation on EGR1 and cFOS expression as well as on EGR and AP1 reporter activity support this prediction. Furthermore, our single cell cFOS expression analysis shows that AP1 activation results indeed from SRF activation. Although we did not analyse EGR expression at the single cell level, we assume that there is a similar link. With respect to cFOS, we show that AII has a major impact on the time course of EGF-induced cFOS expression, resulting in a prolonged upregulation, due to slowed downregulation after induction. Together, the data suggest that EGFR-AT1R synergy lead to a qualitative and temporal change in the transcription regulator pattern and thereby to the synergy at the transcriptome level. IPA^®^ upstream regulator analysis supports this hypothesis predicting a prolonged activity of, e.g. AP1 after 24 h. Furthermore, synergistic AT1R-EGFR-activation is predicted to employ additionally NUPR1, FLI1, and GLI1 as prolonged transcriptional regulators (ST05). Upstream, IPA^®^ pathway analysis identified significant similarities of the 6-h synergy with, e.g. STAT3, BAG2, HIF1α and PI3K/AKT canonical pathways. In addition, TGFB1 is among the mediators of synergistic action, also confirmed by enhanced expression at the protein level. Of note, IPA^®^ pathway analysis indicates the synergistic sustained downregulation of a variety of microRNAs, including miR-149-3p, -185-3p, -328-5p, -361-3p and -762. The underlying mechanism(s) and the possible relevance are unknown at present and have to be addressed in future studies. Further predicted downstream consequences (i.e. cellular impact) of the synergy comprise, as expected, several terms related to cancer but also cardiovascular terms. Several recent studies [4, 35–37] suggest a potential role for AT1R in tumour biology/cancerogenesis. Although AT1R-EGFR crosstalk has not yet been investigated in this context in detail, our data suggest that AT1R-EGFR synergy may play a role also in tumour biology [38]. The predicted consequences include furthermore hypoxia-like response (HIF1α-signalling, VEGF-signalling-pathway), altered carbohydrate metabolism and response to unfolded protein/endoplasmic reticulum stress, indicating that the synergism plays a relevant role for AT1R pathophysiology.

High- and low-affinity binding sites for EGF on living cells have been identified that most probably reflect cytoplasmic domain interactions and different conformational states. The K_D_ values are in the range of *K*_D_ ≈ 0.1–0.3 nmol/l and *K*_D_ ≈ 1–10 nmol/l, respectively [39–41], corresponding to the concentrations used in this study. Furthermore, high- and low-affinity interactions between EGFR and its ligands activate different signalling pathways, with high-affinity sites activating, e.g. ERK1/2 and AKT and low-affinity binding activating, e.g. Signal transducers and activators of transcription and Phospholipase C-gamma 1 [40]. In parallel to the wide range of interaction affinities, determination of in vivo EGF concentrations shows a wide and heterogeneous range depending on different parameters, like tissue or fluid type, species, age, sex, stress or health condition. Values extend from mid-picomolar to moderate nanomolar levels [42–47]. With respect to the cardiovascular system, the alpha-adrenergic-induced increase in plasma EGF from baseline concentration of ≈ 0.2 nmol/l to concentrations of above 5 nmol/l is of relevance. Sympathetic nerve stimulation led to a substantial increase in plasma EGF, too [48]. Furthermore, EGF concentrations in urine and renal tissue are also in the upper range and can exceed plasma levels by a factor of 1000 reaching values above 10 nmol/l [47, 48]. Thus, the concentrations used in our study are in a range comparable to the in vivo situation. Since EGF can be released to act locally (auto- or paracrine) followed by subsequent internalisation and degradation, the effective micromilieu concentrations may be even higher. Our results under coculture conditions reflect the situation during paracrine stimulation and show synergy under these conditions, too. The fact that we observed a synergism not yet at 1 µg/l (≈ 0.15 nmol/l) but at 10 µg/l (≈ 1.5 nmol/l) EGF supports our hypothesis of a relevance under pathophysiological or stress conditions.

As mentioned in the introduction, several cell types express EGFR and AT1R [4], including vascular, cardiac and renal cells, adipocytes, immune cells as well as cells of the central nervous system. Therefore, these cell types are potentially subject to the synergy that we observed at concentrations of EGF and AII occurring in vivo. The relevance of the described synergy results from the qualitative change in signalling pattern during simultaneous AT1R and EGFR activation, resulting in the strong increase of SRF activity (without, e.g. synergistic ERK1/2 activation) that is followed by respective transcriptome alterations (Fig 6D). In cells expressing HB-EGF, simultaneous activation of AT1R and EGFR can be induced by angiotensin II alone in an autocrine fashion, via the release of membrane-bound EGFR ligand. Sole activation of AT1R or EGFR exerts clearly different effects, the sum of which is still different from the observed synergy. Thus, cells can distinguish between direct EGFR action and transactivation and respond with differentiated transcriptional programs. Since SRF is a transcription factor of major importance for the activity of many immediate early genes and thereby participates in cell cycle regulation, apoptosis, cell growth, and cell differentiation, alterations of its activity can have a broad impact, especially for cells of the reno-cardiovascular system. With respect to VSMC, SRF activation via the MRTF pathway is predicted to interfere with cell differentiation, which is relevant during vascular remodelling when VSMC undergo phenotypic switching. Now, the precise nature of this impact in vivo has to be addressed in future studies.

Overall, the effect of AT1R-EGFR synergy on gene expression results from (i) a prolonged activation and (ii) includes the recruitment of additional transcriptional regulatory pathways. Consequently, this supports cellular stress events, thereby inducing dysregulation of tissue homeostasis (parainflammation), a known pathophysiological (and EGFR-dependent) consequence of AT1R overstimulation in the cardiovascular system. Furthermore, AT1R enhances the tumorigenic and proliferative effects of EGFR, which may be part of its recently discussed role in tumour biology.

## Methods

### Cell culture

HK2 (human proximal tubule epithelial cell line, ATCC Cat# CRL-2190, RRID:CVCL_0302) and HEK293 (human embryonic kidney cell line, ATCC Cat# CRL-3216, RRID:CVCL_0063) were obtained from ATCC. Both cell types were cultivated in DMEM/Ham’s F-12 medium (FG 4815, Biochrom, Berlin, Germany), supplemented with 10% foetal calf serum (FCS). Medium was changed to DMEM without FCS prior to addition of stimuli. We used HK2 cells in the initial phase of the study to exclude the possibility that the observed effects on SRF activation is a HEK293 specific phenomenon. Our data show that this is not the case. A7r5 cells (ATCC, ATCC Cat# CRL-1444, RRID:CVCL_0137) were cultivated in DMEM medium. Stable transfected AT1R-HEK293 cells were obtained by cotransfection with pBABEpuro, incubation with 2 µg/ml puromycin and 10 µmol/l losartan (to prevent AT1R activation by residual AII in serum) and limited dilution cloning.

### Next-generation sequencing

Total RNA was isolated as described [49]. Total RNA was isolated by TRIzol Reagent (Invitrogen Life Technologies, Darmstadt, Germany). RNA was washed twice with ethanol and genomic DNA contamination was removed. To do so, one µg of total RNA was treated with 2.0 units of DNase I (RNase-free) (New England Biolabs, Frankfurt am Main, Germany) at 37 °C for 10 min, followed by enzyme inactivation at 75 °C for 10 min for the samples from transiently transfected cells. Samples from stably transfected cells were treated with “Turbo DNAse-free kit” (Invitrogen Life Technologies, Germany) using the “rigorous DNAse treatment” protocol described by the manufacturer and precipitated with 3 M sodium acetate, glycogen and 100% ethanol. The RNA quality was analysed on a 2100 Bioanalyzer (Agilent Technologies GmbH, Waldbronn, Germany). All samples had a RNA Integrity Number (RIN) above 7, whereby RIN 10 indicates maximum quality of the samples.

Paired-end sequencing (2 × 101 or 2 × 150 bp) was performed with HiSeq2000 (Core Unit DNA Technologies of the Faculty of Medicine, University Leipzig, Germany) and NovaSeq6000 (Novogene (UK) Co., Ltd, Cambridge, UK) Illumina systems for samples from transiently and stably transfected, respectively. Libraries were prepared with indexed adapters. Quality control (fastQC, v0.11.3, https://www.bioinformatics.babraham.ac.uk/projects/fastqc/) was performed on the data provided by the service companies. For the data from transiently transfected cells, the adaptors were removed using cutadapt v.1.8.1 [50], while adaptors were already clipped in the data provided by Novogene (UK) Co. for the samples from stably transfected cells. Read mapping was done with Tophat2 (2.0.14, parameters: -r 50 –g 20 –b2-N 1) [51] (human genome *hg19*) and HISAT2 (2.1.0) [52] (*hg38*) for transiently and stably transfected samples, respectively. Counting was done with featureCounts (1.4.6 and 2.0.0) [53]. Genes were annotated using BiomaRt (R package v2.36.1) [54] to access Ensembl archives v75 and v100 for the analyses of transiently and stably transfected samples, respectively.

### Differential expression analysis and gene ontology enrichment analysis

For transiently transfected cells, normalisation was performed using R package EdgeR (3.4.2) [55] from Bioconductor (https://www.bioconductor.org/). The counts were normalised using the “trimmed mean of M values” (TMM) method. A false discovery rate (FDR) of 0.05 was used to determine if genes were significantly regulated. Additional filters such as FPM (fragments per million) > 5 and Fold Change > 1.5 were applied.

For stably transfected cells, differential expression analysis was performed using R packages EdgeR (3.28.1) [55] and DESeq2 (1.26.0) [56] from Bioconductor (https://www.bioconductor.org/). During DESeq2 analysis, genes were filtered for those with at least one normalised count in more than five replicates. The counts were normalised using the “trimmed mean of M values” (TMM) method for edgeR analysis. A false discovery rate (FDR) of 0.01 was used to determine if genes were significantly regulated. Only the genes that were found regulated with both tools (overlap of the analysis outputs) were considered for further analysis steps. In addition, genes with abs(log2 Fold Change) ≥ log2(1.5) and with more than 5 FPM on average (in control samples when downregulated and in treated samples when upregulated) were filtered.

Raw data are publicly available on the GEO (Gene Expression Omnibus) database (https://www.ncbi.nlm.nih.gov/geo). GEO IDs: GSE162770.

Gene ontology enrichment analysis was performed by g:Profiler (http://biit.cs.ut.ee/gprofiler/; [57]). Ingenuity Pathway Analysis (IPA) software (Qiagen, Hilden, Germany) was used for functional analysis (Canonical Pathways, Upstream Regulator and Downstream Effects Analyses; these features are not included in g:Profiler) on the lists of regulated genes (results of the differential expression analyses). Their Ensembl identifiers were mapped to networks available in the software database. For the canonical pathway analysis, enriched pathways were ranked according to how relevant they were for the genes provided as input. Multiple testing was performed using the Benjamini–Hochberg (B–H) procedure. Analyses were corrected for multiple testing as described for the corresponding tools.

### Single-cell reporter gene analysis by digital high-content microscopy

We assessed activity of the transcription factors SRF, EGR1 and AP1 by reporter gene assays. Changes in the expression of the reporter gene are a measure for transcription factor activation. Thus, we measured reporter gene activity under different conditions, calculated the changes versus control and denominated these values transcription factor activation (e.g. SRF activation). Reporters for SRE (sequence GGATGTCCATATTAGGA) or Egr-1 (sequence CGCCCCCGCG), AP1 (sequence TGAGTCAG) transcription factors were purchased from Qiagen, Hilden, Germany. We used the Cignal™ System (http://www.sabiosciences.com/reporterassays.php) with Monster-green fluorescent protein (MGFP) as reporter. The respective transfection control was red fluorescent protein (RFP) under the control of a constitutive CMV promoter. After transfection with Polyfect (Qiagen, Hilden, Germany), cells were incubated as described in the figure legends and reporter activity was determined as recommended by the manufacturer by digital fluorescence microscopy (Cytation 3, BioTek, Bad Friedrichshall, Germany or the PerkinElmer Operetta CLS™ high-content screening system). To determine the cellular responses, first transfected cells were identified according to their red fluorescence (Ex 586/15 nm; Em 647/57 nm; DM 605 nm; LED 590 nm) and their number, mean fluorescence intensity, area, circularity and integral fluorescence intensity determined. Cell identification and determination of the parameters was performed with the Gen5 2.09 software (BioTek, Bad Friedrichshall, Germany). For this purpose, the object recognition parameters (background fluorescence, threshold fluorescence change, rolling ball diameter, object minimum and maximum size, light exposure time, light intensity, gain of image acquisition) were determined during three independent training experiments and subsequently applied to all experiments, making them comparable. Second, the mean green fluorescence intensity (Ex 469/35 nm; Em 525/39; DM 497 nm; LED 465 nm) of the red cells as well as the integral green fluorescence of red cells was determined, using the same routine as for red cells. Finally, red cells that were also green were identified and their number, mean fluorescence intensity, area, circularity and integral green fluorescence integral. The change in fraction of red cells (= transfected cells that could respond) that show a green signal (= active SRF) corresponds to the digital response (switching on of previously inactive cells). The change in green fluorescent intensity of green cells corresponds to the analogue response (enhancing the activity of already activated cells). The overall response to a stimulus is the change in green fluorescence (= SRF activity) of all red cells. This overall response results from the changes in digital and the analogue component (Δoverall = Δdigital x analogue). We compared relative Δdigital and Δanalogue to calculate their contribution. For example: if Δdigital = + 200% and analogue = + 100%, Δdigital is assumed to contribute 2/3 of the overall effect.

Supplementary figure SF01 shows a typical image obtained by digital high throughput microscopy and automated online object analysis. There was reliable identification of transfected cells (red fluorescence from dsRED-construct) and responding cells (green fluorescence from Cignal^®^ SRE-GFP construct). Detailed subanalysis confirmed that all responding cells showed also red fluorescence, indicating that both plasmids were taken up and the coded proteins expressed. This conclusion was confirmed by transfection with constitutive active Cignal^®^ GFP construct together with dsRED. SF01 B shows that virtually all red cells were also positive for green fluorescence. Similar results were obtained for HK2, HEK and A7r5 cells. Transfection with an inactive Cignal^®^ GFP construct resulted in no detectable SRE activity (SF01 B). Whereas the fraction of SRE-positive cells under basal conditions, i.e. cells with an activated SRF-pathway in media free of serum and mediators, was very low (SF01 C), overexpression of EGFR, known to induce strong ligand-independent signalling, resulted in > 80% of SRE-positive cells in relation to transfected cells. Overexpression of the membrane-bound EGFR ligand HB-EGF resulted in > 50% of SRE-positive cells (SF01 C). Overexpression of AT1R (angiotensin II receptor type 1) did not affect basal SRF activity (SF01 C). The number of transfected (= red) cells, their morphology and red fluorescence intensity was stable for at least 48 h (SF01 D). Supplementary figures SF01 E and F show that HEK293 (E) and HK2 cells transfected with the SRE-GFP- and dsRED-constructs only, are not responsive to AII but to EGF. To exclude cell type-specific effects, various experiments were performed in HEK293 and in HK2 cells. Results for HK2 cells are shown as supplementary figures.

### Quantitative RT-PCR

Total RNA was isolated as described above. Reverse transcription (RT) reaction of Dnase-treated total RNA was performed with random primers using a SuperScript II reverse transcriptase (Invitrogen, Life Technologies, Darmstadt, Germany) according to the kit’s manual. Gene expression analysis was carried out as described before [23, 58]. 1 µl cDNA was used in real-time RT-PCR (CFX96 Touch Real-Time PCR Detection System, BioRad, Munich, Germany or 7900HT Fast Real-time PCR system, Applied Biosystems, via Thermo Fisher Scientific, Karlsruhe, Germany). After initial denaturation at 95 °C for 10 min, cDNA was amplified by 40 cycles of 95 °C for 30 s, 60 °C for 30 s, 72 °C for 30 s, followed by 72 °C for 5 min to allow complete extension. Gene expression was normalised to the expression levels of eukaryotic 18S rRNA, CANX and GAPDH. qPCR efficiency was > 90%. The relative mRNA expression of the genes of interest was calculated according to the 2^−ΔΔCt^ method, using the housekeeper signal for normalisation. Each sample was analysed as triplicate. Values are expressed as mean difference between wild type and knockout or between control and stimulation, respectively, ± standard error of mean. The following primers were used (sense, antisense): cFOS (CGGGCTTCAACGCAGACTA, TATCAGTCAGCTCCCTCCTCC), ARC (GGGAGAGTAGAAGTCGCACAC, CTCTGATTCTTCTCCAGGCGG), EGR1 (CAGCACCTTCAACCCTCAG, CAGCACCTTCTCGTTGTTCA), EGR2 (GACAGGAGAGAGTCAGTGGC, TTCTAGGTGCAGAGACGGGA), EGR3 (GGTGACCATGAGCAGTTTGC, TAGGTCACGGTCTTGTTGCC), 18S rRNA (GCATATGCTTGTCTCAAAGA, CCAAAGGAACCATAACTGAT), CANX (ACACAAAAACCCCAAAACGG, CTGGGTCCTCAATTTCACGT) and GAPDH (AAGGTGAAGGTCGGAGTCAA, AATGAAGGGGTCATTGATGG).

### Immunoblotting

For protein expression level determination, cells were lysed with CST lysis buffer (20 mM Tris, pH 7.5 (Illinois Tools Works companies), 150 mM NaCl (Roth), 1% Triton X-100 (Sigma-Aldrich), 1 mM EDTA (Merck), 1 mM EGTA (Sigma-Aldrich), 184 mg/L Na-orthovanadate (Sigma-Aldrich), 2.5 mM Na-pyrophosphate (Sigma-Aldrich), 1 mM β-glycerolphosphate (Sigma-Aldrich)), centrifuged at 13.000 g for 10 min and protein amount was determined with Bradford assay. Equal amounts of the proteins were denaturated with 6 × Laemmli buffer (0.5 M Tris pH 6.8 (Roth GmbH), 10% SDS (Roth), 10% Glycerol (Sigma-Aldrich)) at 95 °C for 5–10 min. Proteins were separated by 10% sodium dodecyl sulphate–polyacrylamide gel electrophoresis (SDS-PAGE) and transferred onto a nitrocellulose membrane. After blocking with 5% nonfat dry milk powder in Tris-buffered saline with Tween20 (TBS-Tween) (20 mM Tris base, pH 7.4 (Illinois Tools Works companies), 150 mM NaCl, 0.05% Tween-20 (Sigma-Aldrich)) membranes were incubated with first antibody diluted in 5% bovine serum albumin (BSA) in TBS-Tween overnight. Horse radish peroxidase (HRP)-coupled secondary antibodies, 1:1000 in 5% nonfat dry milk powder in TBS-Tween) were used. After removal of unbound secondary antibody, three washing steps in TBS-TWEEN were performed. Finally Clarity™ Western ECL Substrate (Bio-Rad, Munich, Germany) was added and the peroxidase activity-based light emission was recorded by an imaging system (Image Quant LAS4000, GE Health care, Buckinghamshire, GB). The following antibodies were purchased from Cell Signaling Technologies, Frankfurt, Germany (phosphoERK1/2 #9101, RRID:AB_331646, 1:1000; ERK1/2 #4696, RRID:AB_390780, 1:1000; phospho-EGFR^Y1068^-XP #3777, RRID:AB_2096270, 1:200; EGFR XP #4267, RRID:AB_2246311, 1:1000; SRF #5147, RRID:AB_10694554, 1:1000; cFOS #2250, RRID:AB_2247211, 1:1000; cJUN #9165, RRID:AB_2130165, 1:1000; EGR1 #4154, RRID:AB_2097035, 1:1000; HSP90 #4874, RRID:AB_2121214, 1:1000; MRTF-A #14760, RRID:AB_2798598, 1:1000; MRTF-B #14613, RRID:AB_2798539, 1:1000; Elk-1 #9182, RRID:AB_2277936, 1:1000; phospho-Elk-1 #9186, RRID:AB_2277933, 1:1000; cSRC #2191, RRID:AB_2196189, 1:1000; phospho-cSRC^Y416^ #6943, RRID:AB_10013641, 1:1000; TGFß #3711, RRID:AB_2063354, 1:1000; ß-actin #3700, RRID:AB_2242334, 1:1000; GAPDH #2118, RRID:AB_561053, 1:1000; anti-rabbit-HRP #7074, RRID:AB_2099233; 1:1000). ARC (1:1000, ab183183, RRID:AB_2756512) and myocardin (1:1000, ab107301, RRID:AB_11128102) antibodies were purchased from abcam (Cambridge, UK), HB-EGF (1:1000, AF-259-NA, RRID:AB_354429) antibody from R&D Systems (Bio-Techne, Wiesbaden-Nordenstadt, Germany). Densitometry analysis was performed with Quantity One^®^ software from BioRad (Feldkirchen, Germany) and the relative expression values calculated under consideration of ß-actin or GAPDH expression as housekeepers.

### EGFR trafficking and immunofluorescence high-content imaging by digital microscopy

The PerkinElmer Operetta CLS™ high-content screening system was used for automated subcellular fluorescence imaging to determine EGFR distribution or co-occurrence of SRE-reporter activation and AP1 expression at the single cell level and its alteration by EGF and AII. For this purpose, HK2 and HEK293 cells were transfected in 60 mm Petri dishes with AT_1_R and EGFR-EGFP or SRE-Reporter, respectively, using Polyfect (Qiagen, Hilden, Germany). 24 h later, cells were harvested and seeded into pre-coated (Poly-l-lysine 0.1 mg/ml) glass bottom 96 well plates. When EGFR distribution was analysed, nuclei were stained using Hoechst 33342 (5 µg/ml) and the first measurement was performed followed by application of either 10 µg/l EGF, 1 nM AngII, or both. Digital microscopy was performed using a 40 × water immersion objective. Automated cell finding and identification routine with a 20 × objective was preceded.

To investigate the co-occurrence of SRE-reporter activation and AP1 expression, cells were stimulated as indicated above and incubated for 6 h. After cell fixation with 4% formaldehyde for 72 h at 4 °C, cells were permeabilized (0.1% Triton X-100 in TBS; 37 mg/l Na-orthovanadate) and blocked using 5% donkey serum in permeabilization buffer. cFOS primary antibody (Cell Signaling Technology Cat# 2250, RRID:AB_2247211, Frankfurt, Germany) was diluted 1:1000 in 1% BSA in permeabilization buffer and incubated overnight at 4 °C. Donkey anti-rabbit AlexaFluor568 secondary antibody (#A10042, Invitrogen Life Technologies, Darmstadt, Germany) was then diluted 1:500 in 1% BSA in permeabilization buffer and incubated for 1 h in the dark at room temperature. Nuclei were stained by diluting DAPI in PBS at 1 µg/ml and applied for 1 min in the dark at room temperature. Digital microscopy was performed using a 20 × objective. Subsequently, the images were analysed with the Harmony software and in-build routines after adjusting the necessary parameters.

### Proximity labelling mediated by engineered ascorbic acid peroxidase (APEX)

Proximity labelling was performed according to Paek et al. [32]. Cells were transfected with pcDNA5/FRT/TO-AT1R-APEX (0.5 µg/75 cm^2^) or pcDNA3-APEX2-NES (control for non-specific interaction) at 80% confluence using Lipofectamine. After 48 h, medium was replaced by labelling medium (DMEM without serum plus 500 µM biotinyl tyramide (Sigma) for 1 h. Subsequently, cells were incubated for 30 min with 1 nmol/l AII, 10 µg/l EGF, AII + EGF or control followed by 1 min exposure to 1 mmol/l H_2_O_2_. Thereafter, cells were washed three times with a quenching solution (Dulbecco’s phosphate buffered saline + 10 mmol/l sodium ascorbate, 5 mmol/l trolox and 10 mmol/l sodium azide). Cell were scratched of the dishes (in the presence of 5 mmol/l EDTA), centrifuged, washed twice with quenching solution and lysed in CST lysis buffer (see above) for 30 min at 4 °C. After sonication (3 × 15 s), the samples were centrifuged for 10 min (13.000 rpm, 4° C) and the supernatant used for pull-down. Pull-down was performed with 1 mg MyOne™ Streptavidin T1 Dynabeads (Invitrogen, according to the manufacturers’ protocol) per 400 pmol of peptide. Samples were mixed with the beads and incubated for 30 min at room temperature while carefully rotating. Subsequently, the vial was placed in the magnet holder and the supernatant removed after 3 min. After three washing steps, the samples were incubated with Laemmli buffer (see above) at 95 °C for 5 min and used for immunoblotting.

### Materials

The following plasmids were used: pCMV6-Myc-DDK-HB-EGF (RC 207688, Origene, Rockville, MD), pCMV6-XL4-AT1R (SC 108918, Origene, Rockville, MD); pEGFR-EGFP (RRID:Addgene_32751, addgene, Watertown, MA; Carter et al. J Biol Chem. 1998 Dec 25;273(52):35000–7), pDsRed2 (#632406, Clontech, Mountain View, CA), pBABEpuro [59], pcDNA5/FRT/TO-AT1R-APEX (RRID:Addgene_96847, addgene; Paek et al. Cell. 2017 Apr 6;169(2):338–349), pcDNA3 APEX2-NES (RRID:Addgene_49386, addgene).

Unless stated otherwise, all materials were purchased from Sigma, Munich, Germany.

### Statistics

ANOVA followed by post hoc testing, Student’s *T* test or Mann–Whitney rank sum test were used as applicable according to pre-test data analysis by SigmaPlot 12.5. A *p* value < 0.05 was considered significant. Biometrical planning was performed with *α* = 0.05 and *β* = 0.8

## Supplementary Information

Below is the link to the electronic supplementary material.

Supplementary file1 (XLSX 378 KB)

Supplementary file2 (XLSX 1034 KB)

Supplementary file3 (XLSX 3231 KB)

Supplementary file4 (XLSX 3888 KB)

Supplementary file5 (XLSX 1010 KB)

Supplementary file6 (XLSX 617 KB)

Supplementary file7 (PDF 2531 KB)

## Data Availability

Datasets generated during and/or analysed during the current study are available in the gene expression omnibus database with the study identity GSE162770. (https://www.ncbi.nlm.nih.gov/geo/query/acc.cgi?acc = GSE162770). All further data generated or analysed during this study are included in this published article and its supplementary information files.

## Abbreviations

ADAM: A disintegrin and metalloproteinase
AII: Angiotensin II receptor type 1
AP1: Activator protein 1
APEX: Ascorbic acid peroxidase
ARC: Activity-regulated cytoskeleton-associated protein
AT1R: Angiotensin II receptor type 1
BIM: Bisindolylmaleimide
BSA: Bovine serum albumin
CANX: Calnexin
cFos: Fos proto-oncogene, AP1 transcription factor subunit
cJun: Jun proto-oncogene, AP1 transcription factor subunit
cSRC: Cellular SRC proto-oncogene, non-receptor tyrosine kinase
DAPI: 4′,6-Diamidin-2-phenylindol
DMEM: Dulbecco's modified eagle medium
ECL: Enhanced chemiluminescence
EGF: Epidermal growth factor
EGFR: Epidermal growth factor receptor
EGR: Early growth response protein
Elk-1: ETS like-1-protein
ER: Endoplasmic reticulum
ErbB: Erythroblastic leukaemia viral oncogene
ERK1/2: Extracellular signal-regulated kinases 1/2
FCS: Foetal calf serum
FDR: False discovery rate
FPM: Fragments per million
GAPDH: Glyceraldehyde-3-phosphate dehydrogenase
GEO: Gene expression omnibus
GFP: Green fluorescent protein
GO: Gene ontology
GPCR: G-protein-coupled receptor
Grb2: Growth factor receptor-bound protein 2
HB-EGF: Heparin-binding EGF-like growth factor
hg: Human genome
HRP: Horse radish peroxidase
HSP90: Heat shock protein 90
MAPK: Mitogen-activated protein kinase
MEK1/2: Mitogen-activated protein kinase kinase 1/2
miR: Micro-RNA
MRTF: Myocardin-related transcription factor
NFkB: Nuclear factor 'kappa-light-chain-enhancer' of activated B cells
PKC: Protein kinase C
PMA: Phorbol-12-myristat-13-acetat
PP2: PP2 (Src-family inhibitor)
Rho: Ras homologue
RIN: RNA integrity number
ROCK: Rho-kinase
SDS: Sodium dodecyl sulphate
SRE: Serum response element
SRF: Serum response factor
TBS: Tris-buffered saline
TCF: Ternary complex factor
TGFß: Transforming growth factor beta
TMM: Trimmed mean of M values
