# Supplementary material for: Synergy of epidermal growth factor (EGFR) and angiotensin II (AT1R) receptor determines composition and temporal pattern of transcriptome variation
Source: Cell Mol Life Sci. 2021 Dec 18;79(1):57. doi: 10.1007/s00018-021-04065-5 (PMC8738452; doi:10.1007/s00018-021-04065-5)

Supplementary figues

Supplementary figure SF01A-F. Establishment of the method for single cell reporter gene analysis. A) Representative photomicrographs of trasfected cells (red) and SRF-postive cells (green). B) Quantitative analysis of positive (contitutive active promoter) and negative (without promoter) controls. N=12. C) Effect of transfection on basal SRF-activity. N=18. D) Stability of cell number, size, morphology and RFP-signal over time under control conditions. N=18. E and F) Mock-transfected HEK293 and HK-2 cells are responsive to EGF but not to AII, in agreement with endogenous EGFR expression but a lack of AT1R expression. N=18.

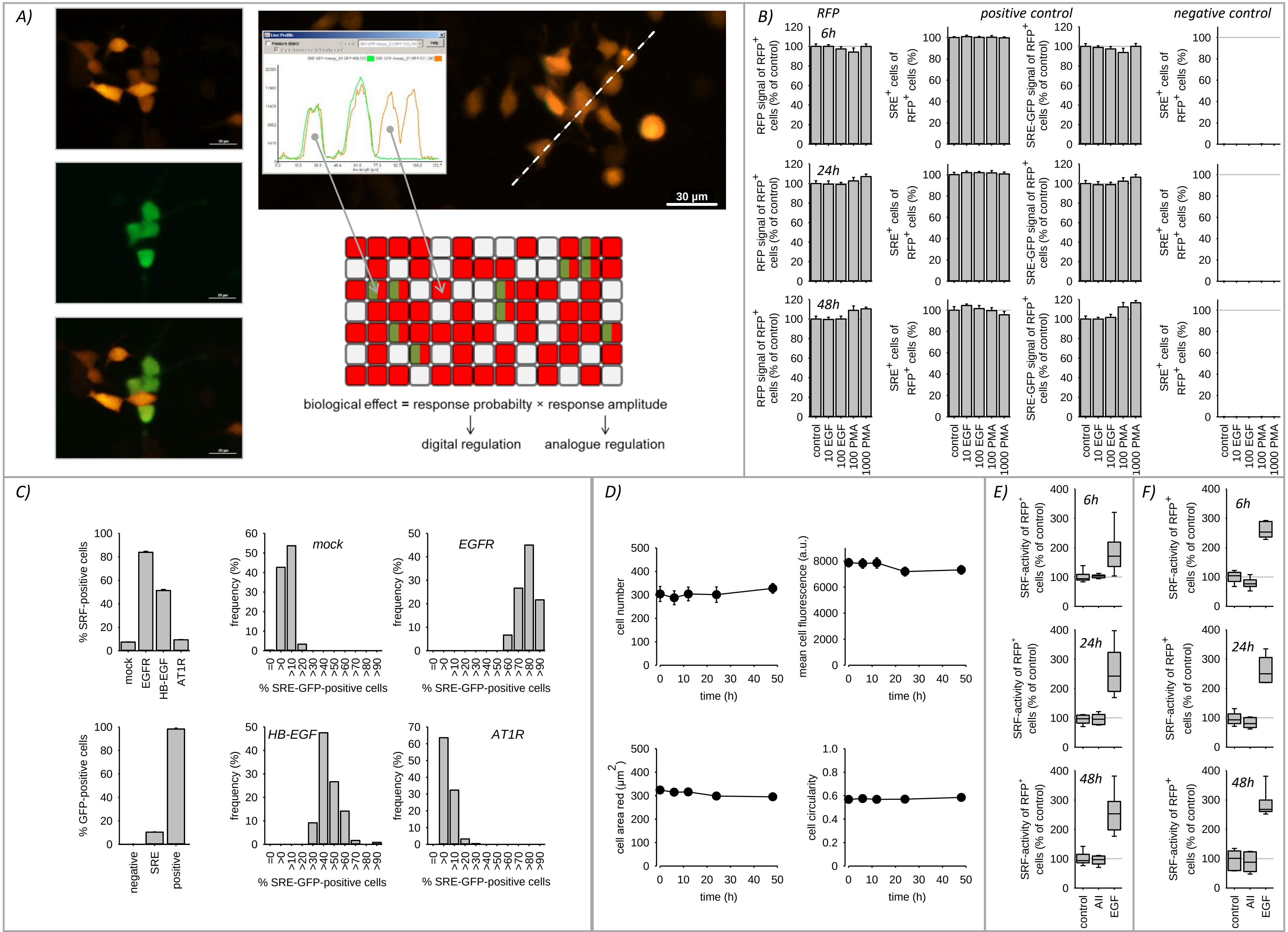

Supplementary figure SF01G. Analysis routine and parameters during single cell gene reporter assays.

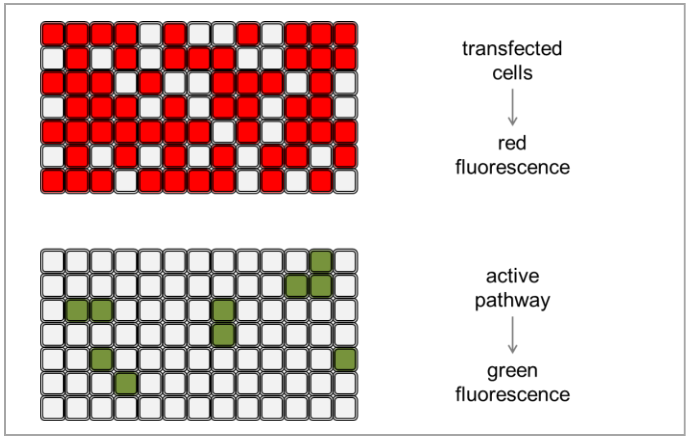

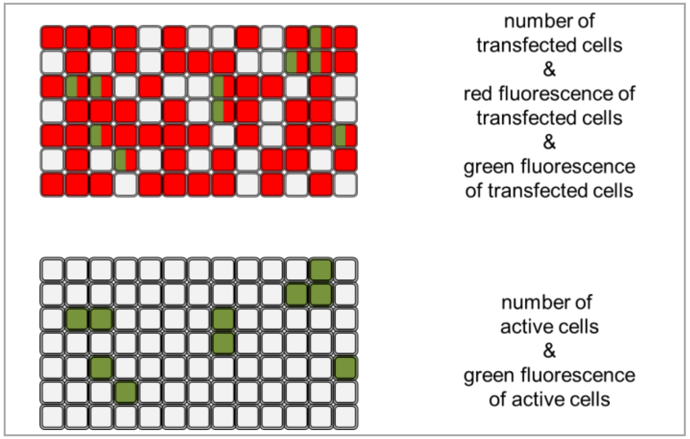

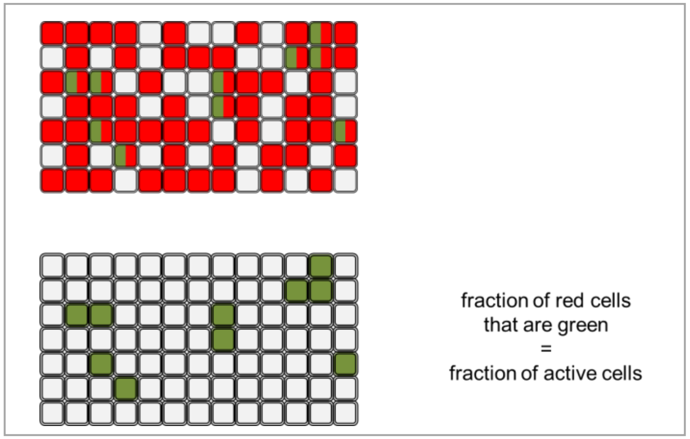

measure	biology
red cell number	transfection control
red cell intensity	stability control | protein synthesis rate
green intensity of red cells	gross effect
green intensity of green cells	analogue regulation
number of green cells	digital regulation
red cell area	hypertrophy
red cell circulartity	shape changes
green cell circularity	shape changes related to signalling

Δ green intensity of RFP^{+} cells $= \Delta$ mean green intensity $\times \Delta$ size

$= \Delta$ integral green of RFP^{+} cells

Δ green intensity of GFP^{+} cells $= \Delta$ mean green intensity $\times \Delta$ size

$= \Delta$ integral green of GFP^{+} cells

Supplementary figure SF02. A) Time-course of SRF activation by EGF (10 µg/l) in HEK293 cells. N=30. B) Concentration-response curve of SRF activation by EGF after 24h in HEK293 cells. t=24h. N=24. C-G) Pharmacological identification of pathways involved in EGFR-induced SRE-activation after 24h. N=24. C) Inhibition of EGFR-kinase by 100 nmol/l AG1478, D) ERK1/2-phosphorylation by 1 µmol/l U0126, E) actin polymerization by 100 nmol/l latrunculin B (LatB), F) Rho-kinase (ROCK) activity by 10 µmol/l Y27632 and G) protein kinase C activity by 100 nmol/l bisindolylmaleimide (BIM). H) Time-course of SRF activation by PMA (1 µmol/l) in HEK293 cells. N=24. I) Concentration-response curve of SRF activation by EGF in HEK293 cells. N=18. J) Inhibition of EGFR-kinase by 100 nmol/l AG1478 (N=18), D) ERK1/2-phosphorylation by 1 µmol/l U0126 (N=18), E) actin polymerization by 100 nmol/l latrunculin B (LatB; N=18), F) Rho-kinase (ROCK) activity by 10 µmol/l Y27632 (N=24) and G) protein kinase C activity by 100 nmol/l bisindolylmaleimide (BIM, N=24). * = p<0.05 versus control, if not indicated otherwise.

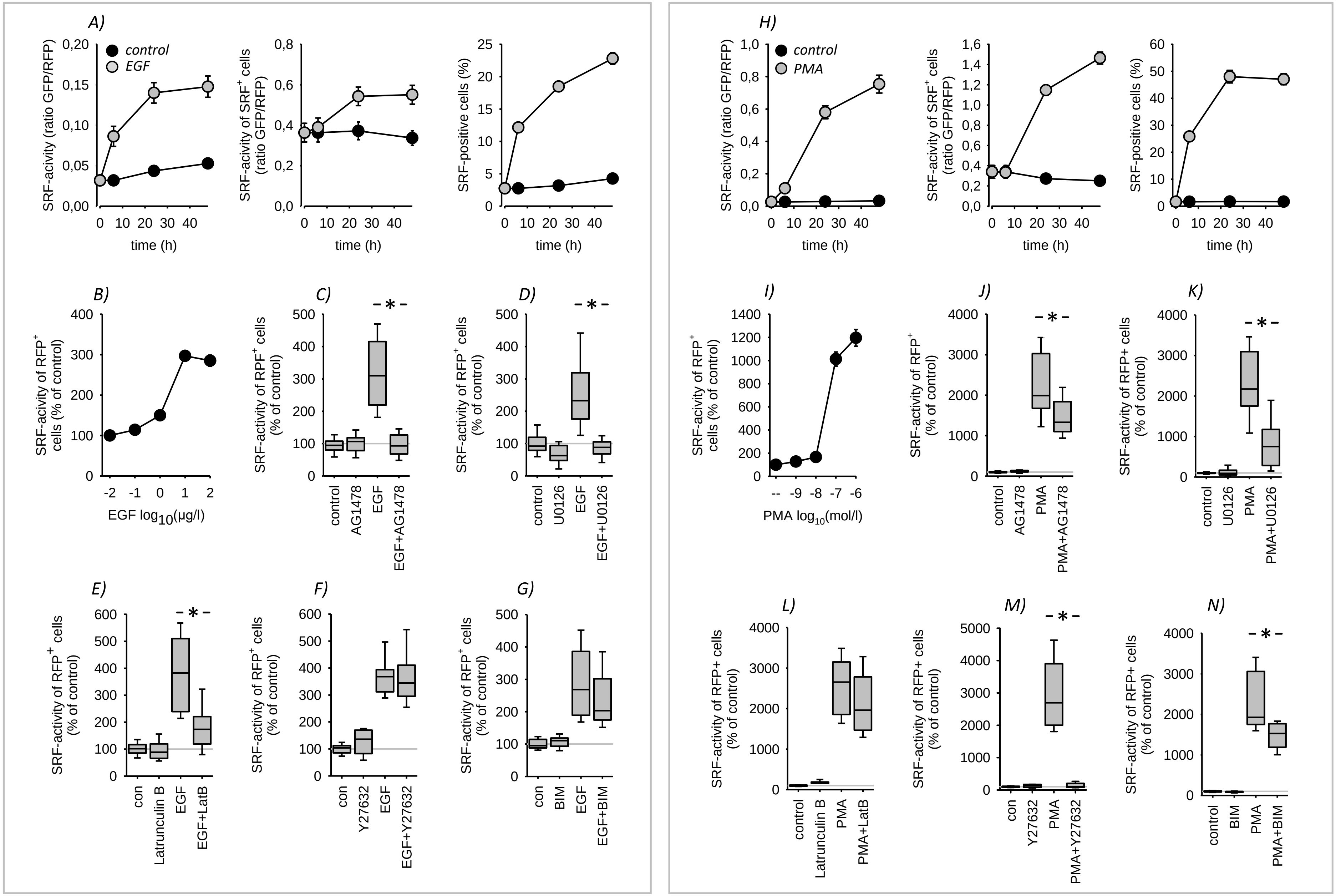

Supplementary figure SF03. A) Time-course of SRF activation by EGF (10 µg/l) in HK-2 cells. N=30. B) Concentration-response curve of SRF activation by EGF in HEK293 cells. t=24h. N=12. C-G) Pharmacological identification of pathways involved in EGFR-induced SRE-activation. t=24h. N=24. C) Inhibition of EGFR-kinase by 100 nmol/l AG1478, D) ERK1/2-phosphorylation by 1 µmol/l U0126, E) actin polymerization by 100 nmol/l latrunculin B (LatB), F) Rho-kinase (ROCK) activity by 10 µmol/l Y27632 and G) protein kinase C activity by 100 nmol/l bisindolylmaleimide (BIM). * = p<0.05. H) Time-course of SRF activation by PMA (1 µmol/l) in HEK293 cells. N=18. I) Concentration-response curve of SRF activation by EGF in HEK293 cells. N=18. J) Inhibition of EGFR-kinase by 100 nmol/l AG1478 (N=18), D) ERK1/2-phosphorylation by 1 µmol/l U0126 (N=18), E) actin polymerization by 100 nmol/l latrunculin B (LatB; N=18), F) Rho-kinase (ROCK) activity by 10 µmol/l Y27632 (N=18) and G) protein kinase C activity by 100 nmol/l bisindolylmaleimide (BIM, N=24). * = p<0.05 versus control, if not indicated otherwise.

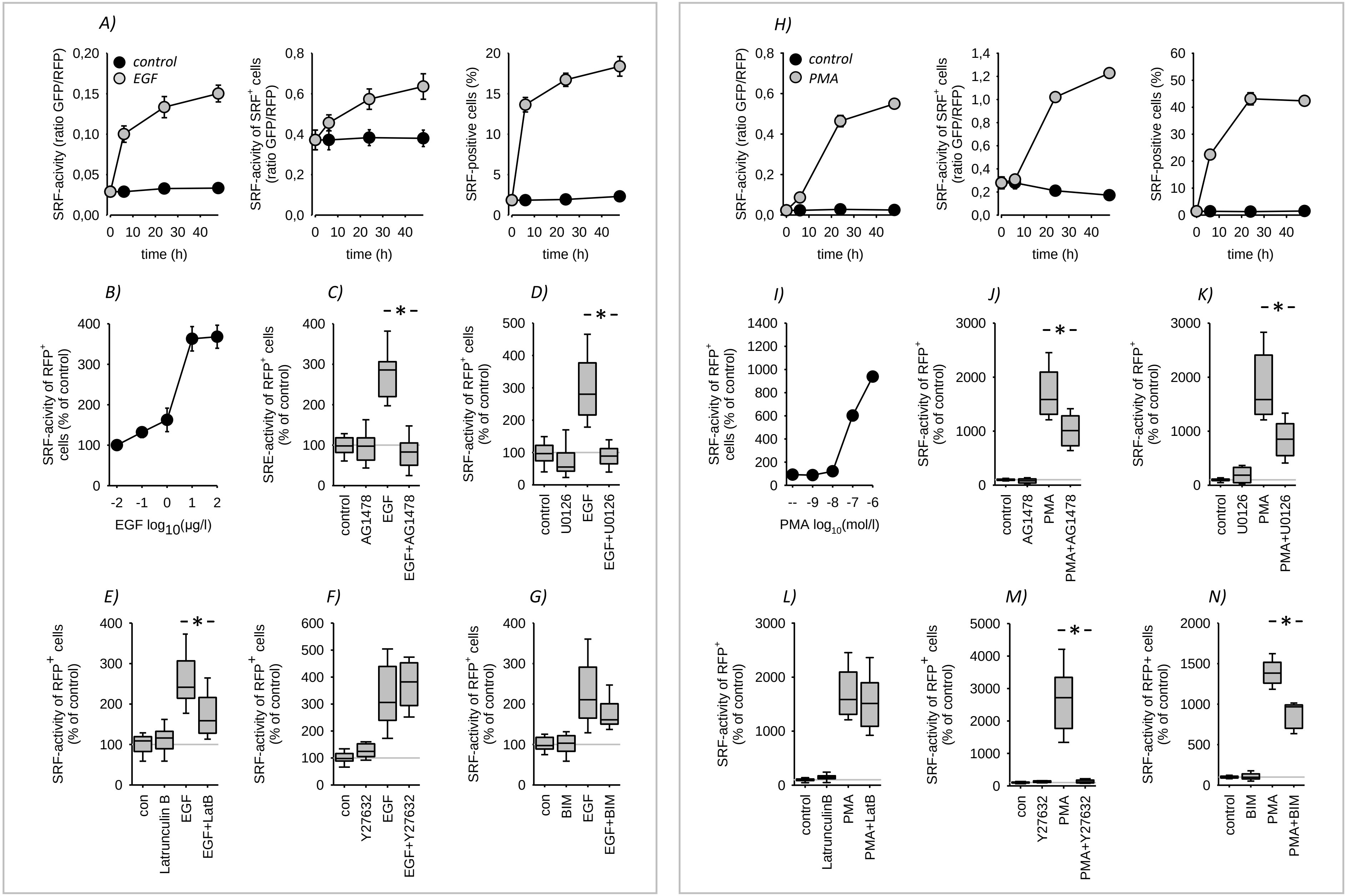

Supplementary figure SF04. Pharmacological identification of pathways involved in AT1R-induced SRE-activation by angiotensin II (1 nmol/l AII) in HEK293 cells transfected with AT1R. t=24h. N=24. A) Inhibition of EGFR-kinase by 100 nmol/l AG1478, B) ERK1/2-phosphorylation by 1 µmol/l U0126, C) actin polymerization by 100 nmol/l latrunculin B (LatB), D) Rho-kinase (ROCK) activity by 10 µmol/l Y27632 and E) protein kinase C activity by 100 nmol/l bisindolylmaleimide (BIM). * = p<0.05.

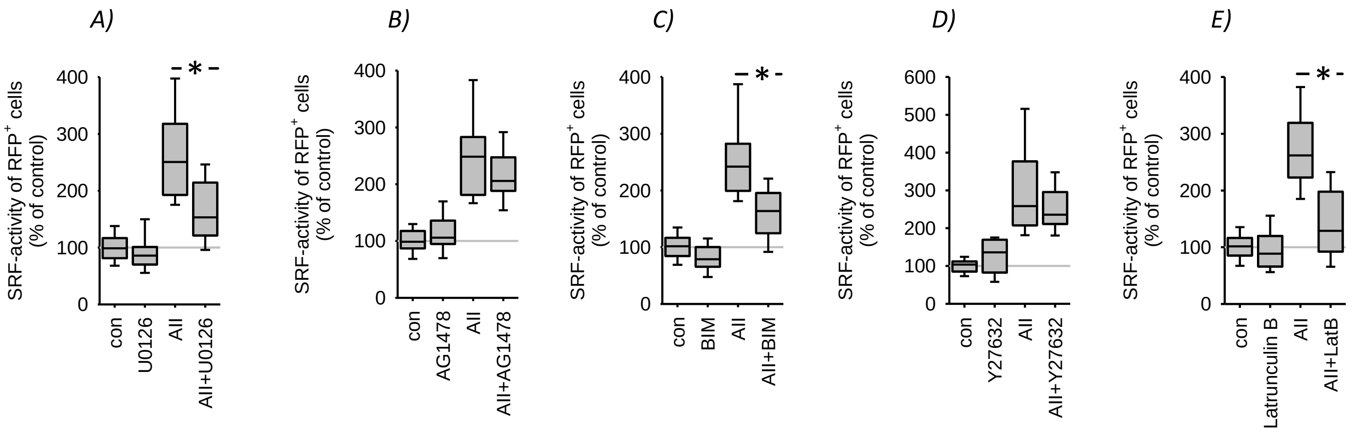

Supplementary Figure SF05. A) Synergistic effect on aggregate SRF-activity in HEK293 cells transfected with AT1R. B) Synergistic effect on SRF-activity of SRF-positive cells (analogue effect). C) Synergistic effect on the fraction of SRF-positive cells (digital effect). D)-F) EGFR-AT1R-synergism depends on the EGF concentration. t=6, 24, 48h. N=24. * = p<0.05 versus control, if not indicated otherwise.

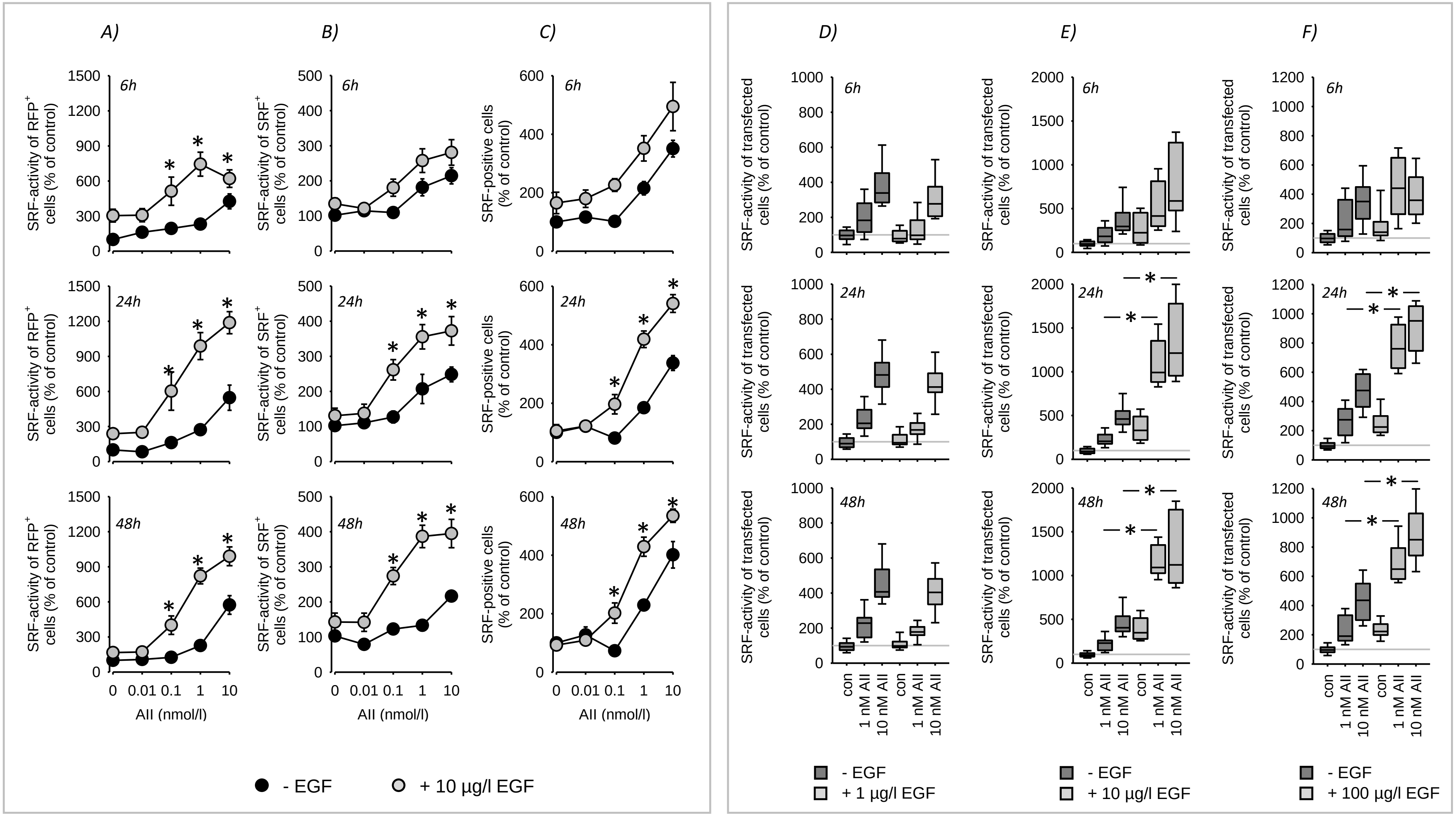

Supplementary Figure SF06. A) Time-course of SRF activation by angiotensin II (1 nmol/l AII) in HK-2 cells transfected with AT1R. N=24. B) Concentration-response curve of SRF activation by AII in HK-2-AT1R cells after 24h. t=24h. N=18. C-G) Pharmacological identification of pathways involved in AT1R-induced SRE-activation. t=24h. N=24. C) Inhibition of EGFR-kinase by 100 nmol/l AG1478, D) ERK1/2-phosphorylation by 1 µmol/l U0126, E) actin polymerization by 100 nmol/l latrunculin B (LatB), F) Rho-kinase (ROCK) activity by 10 µmol/l Y27632 and G) protein kinase C activity by 100 nmol/l bisindolylmaleimide (BIM). H) Synergistic action of 10 µg/l EGF and AII on SRF-activity. t=6, 24 or 48h. N=18. I) The action of 1 nmol/l AII and the synergistic action of 1 nmol/l AII + 10 µg/l EGF are prevented by the AT1R blocker losartan (L, 1 µmol/l). t=6, 24 or 48h. N=12). J) Estimation of the maximum effect (Emax) elicited by AII in the absence and presence of EGF and of the half-maximum AII concentration (EC50) from the data in panel H. * = p<0.05 versus control, if not indicated otherwise.

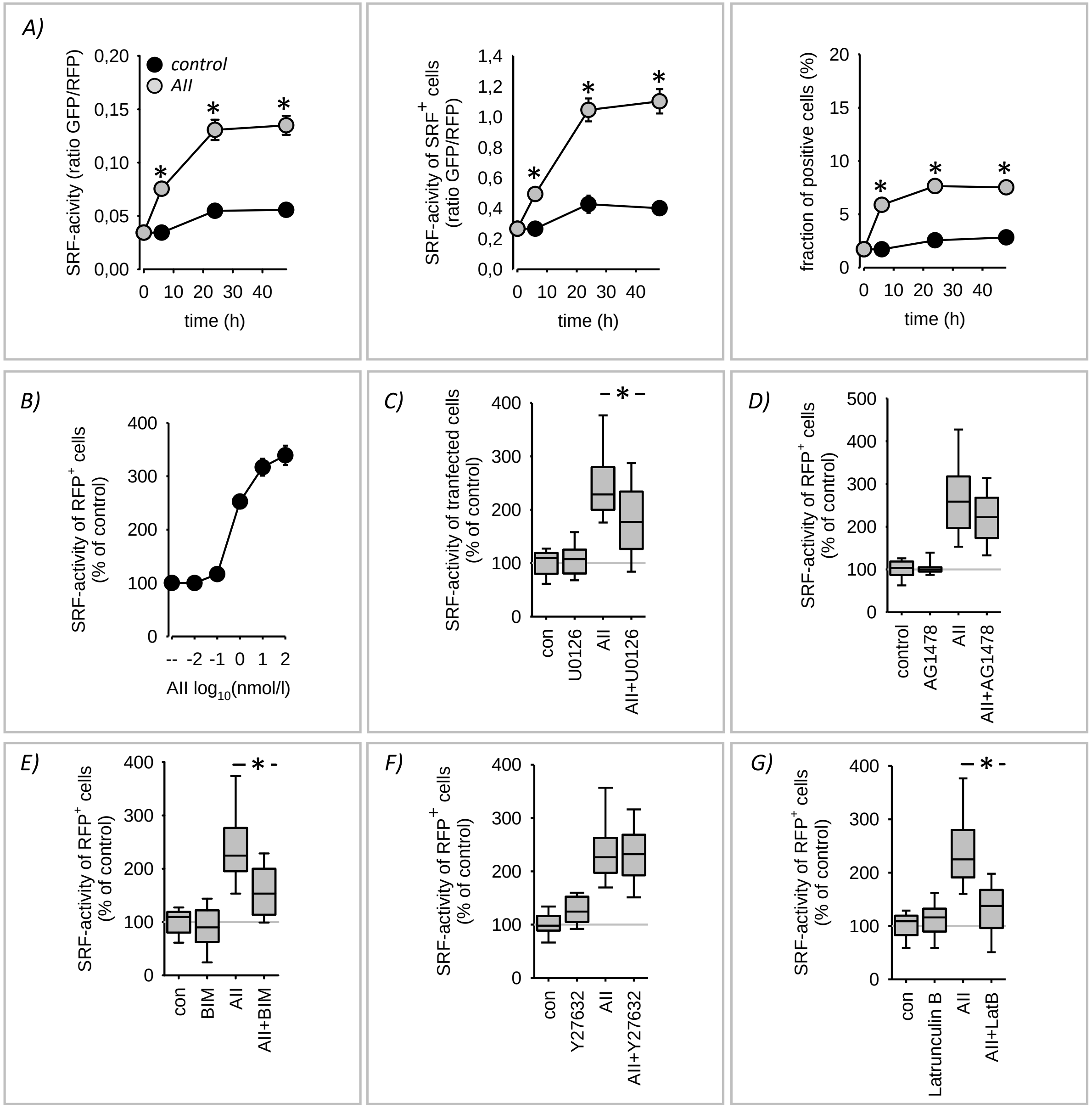

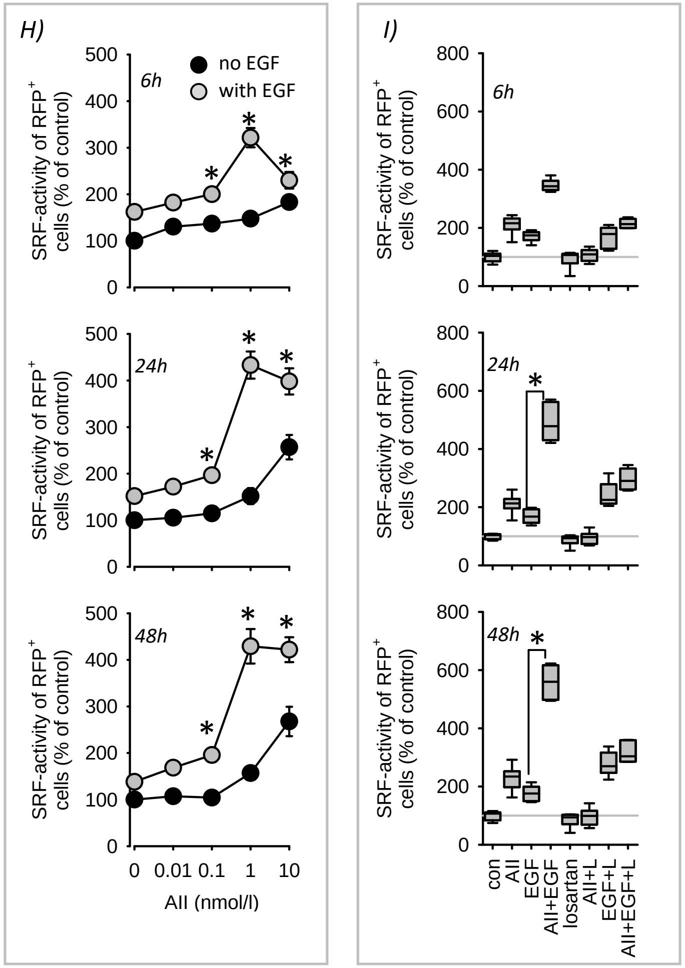

J)

24h	+ EGF	- EGF
Emax	288±25	167±18
EC50	0.24±0.09	1.9±0.9

48h	+ EGF	- EGF
Emax	313±26	181±22
EC50	0.26±0.11	1.7±1.1

Supplementary figure SF07. A) Synergistic effect on aggregate SRF-activity in HK2 cells transfected with AT1R. B) Synergistic effect on SRF-activity of SRF-positive cells (analogue effect). C) Synergistic effect on the fraction of SRF-positive cells (digital effect). D)-F) EGFR-AT1R-synergism depends on the EGF concentration. t=6, 24 or 48h. N=24. * = p<0.05 versus control, if not indicated otherwise.

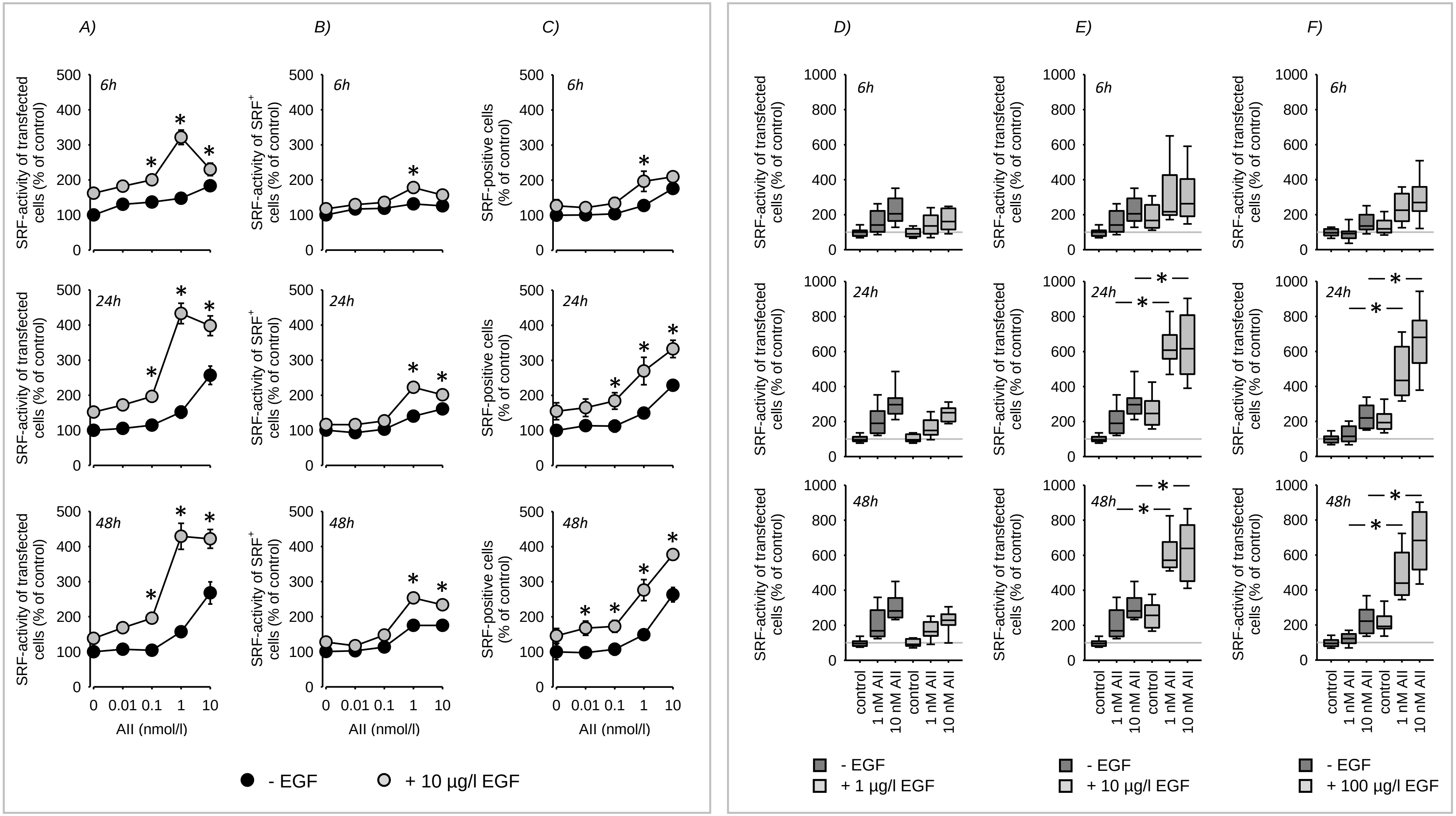

Supplementary figure SF08. A) HB-EGF is not expressed in wt HEK-293 cells (lane). Transfection with HB-EGF leads to detectable HB-EGF expreesion (lanes 1 and 2). B) Dose-dependent increase in SRF-activity after transfection of HEK-AT1R cells with HB-EGF. Values were obtained 48 h after transfection. N=18.

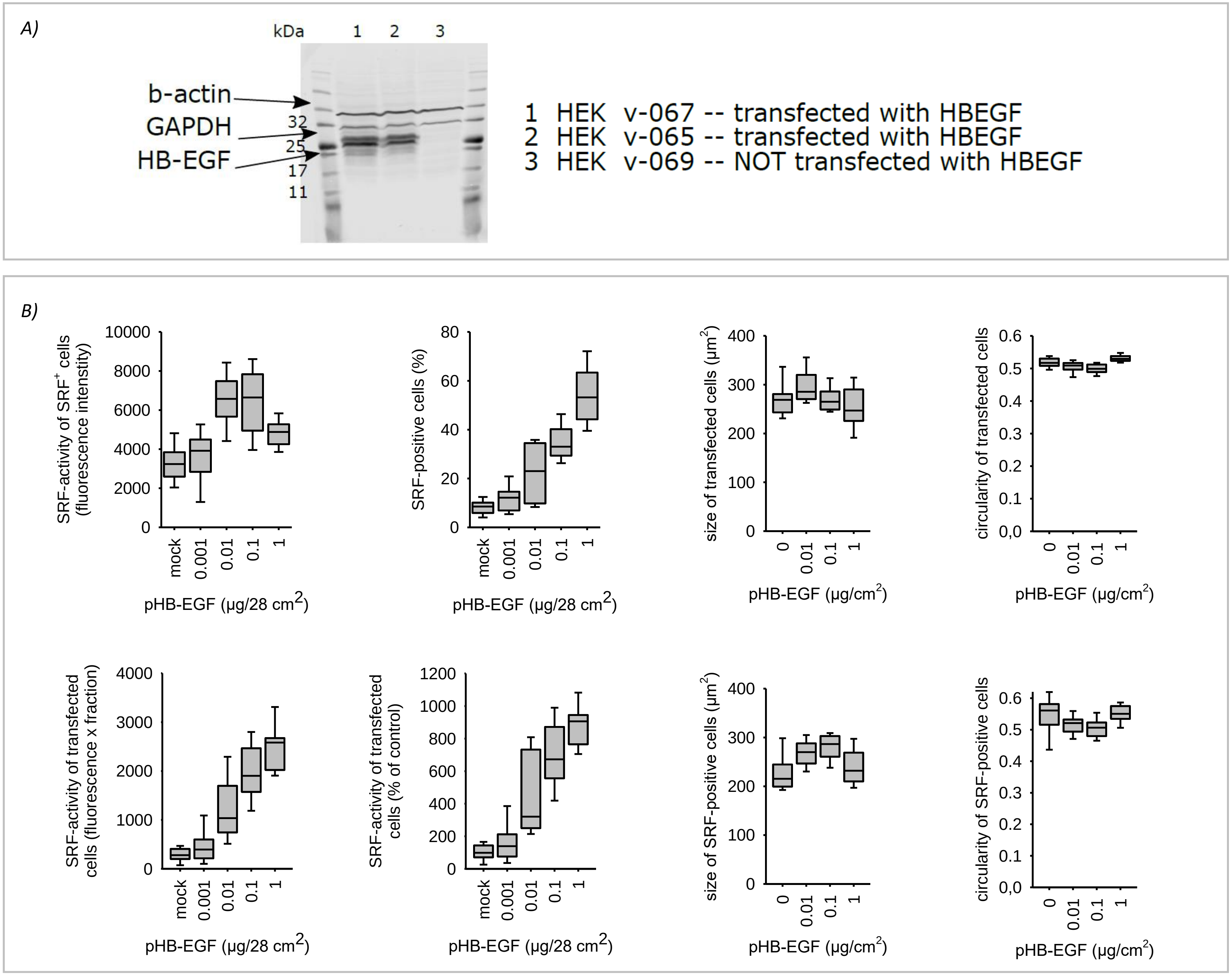

Supplementry Figure SF09. A) In the absence of HB-EGF AT1R-induced activation of SRF is independent of EGFR. HB-EGF expression leads to additional EGFR-mediated SRF-activation by AT1R. B) AT1R-induced activation of SRF normalized to control values in the absence of HB-EGF. HB-EGF expression leads to an increase in maximum SRF-activation and tincreases the AII-sensitiviy (EC50 values). t=6, 24 or 48h. N=18. * = p<0.05 versus control, if not indicated otherwise.

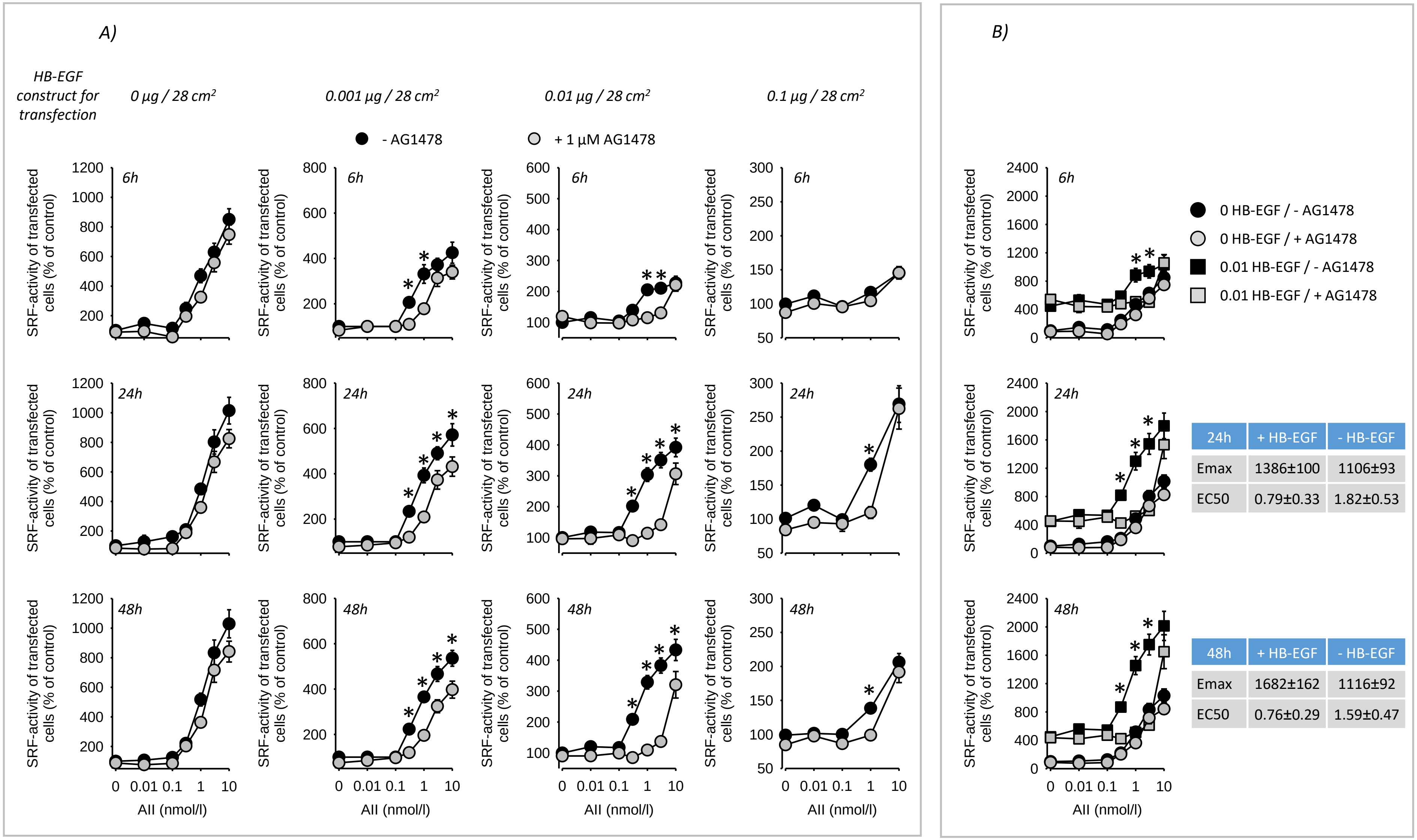

24h	+ HB-EGF	- HB-EGF
Emax	1386±100	1106±93
EC50	0.79±0.33	1.82±0.53

48h	+ HB-EGF	- HB-EGF
Emax	1682±162	1116±92
EC50	0.76±0.29	1.59±0.47

Supplementry Figure SF10. Coculture of reporter cells (transfected with SRE.GFP) with HB-EGF-tranfected „donor“ cells shows that paracrine HB-EGF signalling also leads to a synergistic effect of AT1R and EGFR with respect to SRF activation. A) HB-EGF expressing donor cells without AT1R induce no AII-sensitivity in reporter cells. B) Donor cells with HB-EGF and AT1R induce a moderate AII-sensitivity of reporter cells mediated by EGFR activation. C) Additional expression of AT1R in reporter cells leads to synergistic (EGFR-dependent) SRF activation. D) When AT1R is expressed in reporter but not in donor cells, the effect of AII is EGFR-independent. E) This effect is similar to the reaction of reporter cells in monoculture. 1 µmol/l AG1478; 0.1, 1 or 10 nmol/l AII. t=6, 24 or 48h. N=18. * = p<0.05 versus control, if not indicated otherwise.

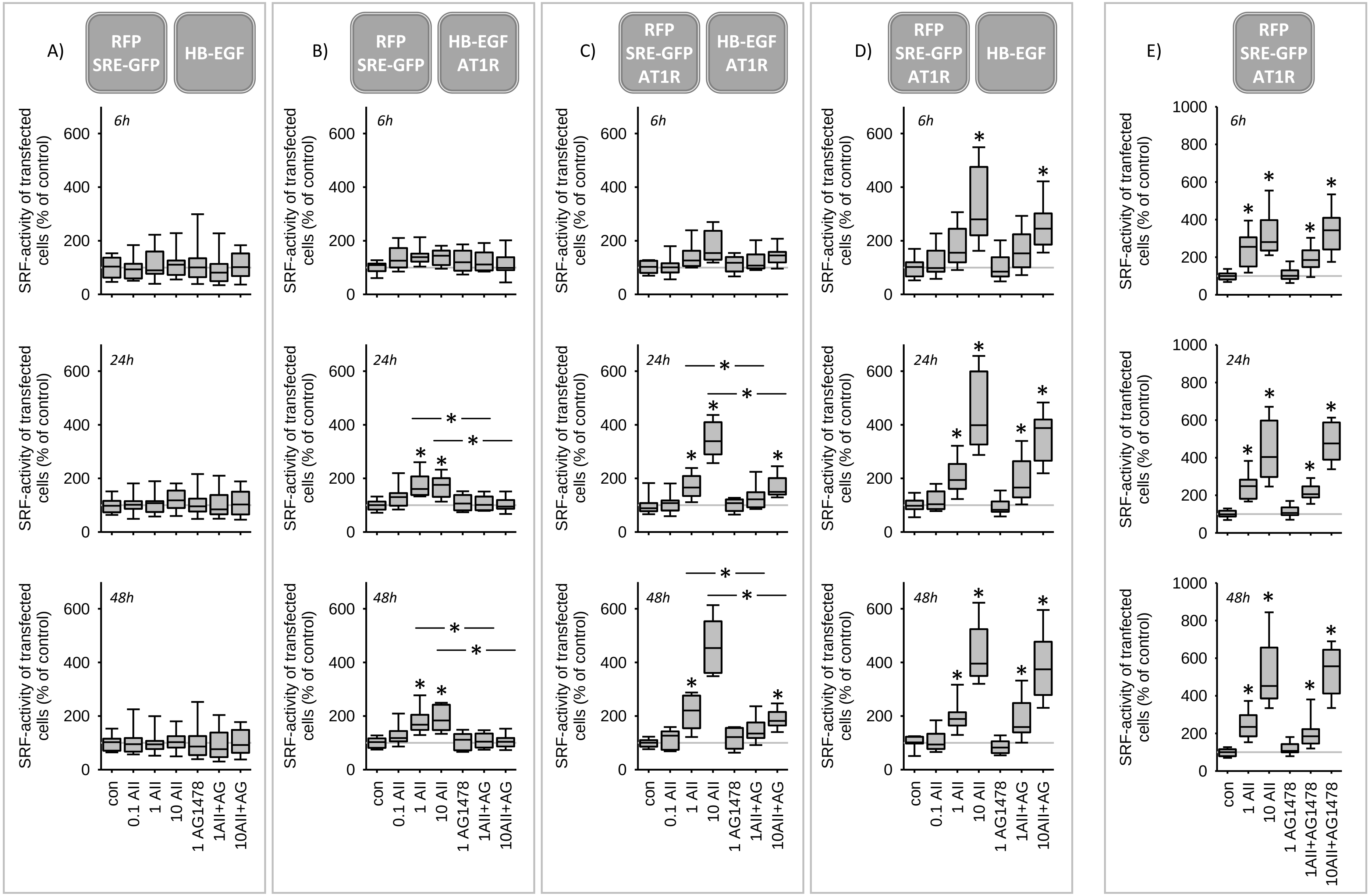

Supplementry Figure SF10. F) Control SRF-activity and fraction of SRF-positive cells under control conditions for the different coculture scenarios. N=12. . * = p<0.05 versus con & AT1R-HB-EGF.

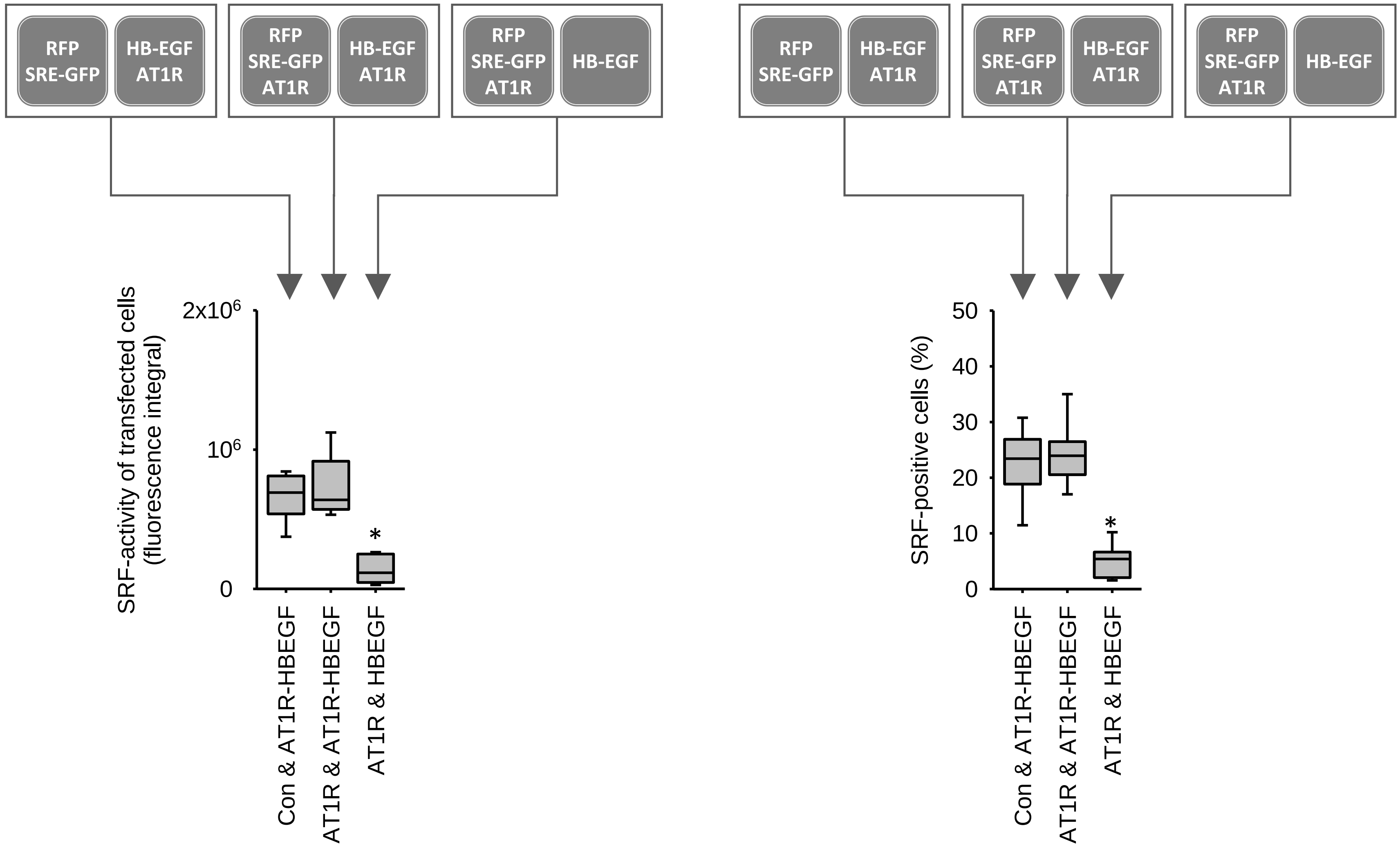

Supplementary Figure SF11. A) The synergism of AII and EGF is also observed in vascular smooth muscle cells (A7r5 cell line). N=24. B) representative photomicrographs of A7r5 cells (red = transfected cells, green = cells with active SRF). N=24. C) Inhibition of SRF activation by EGFR-inhibition (100 nmol/l AG1478). t=6, 24 or 48h. N=12. * = p<0.05.

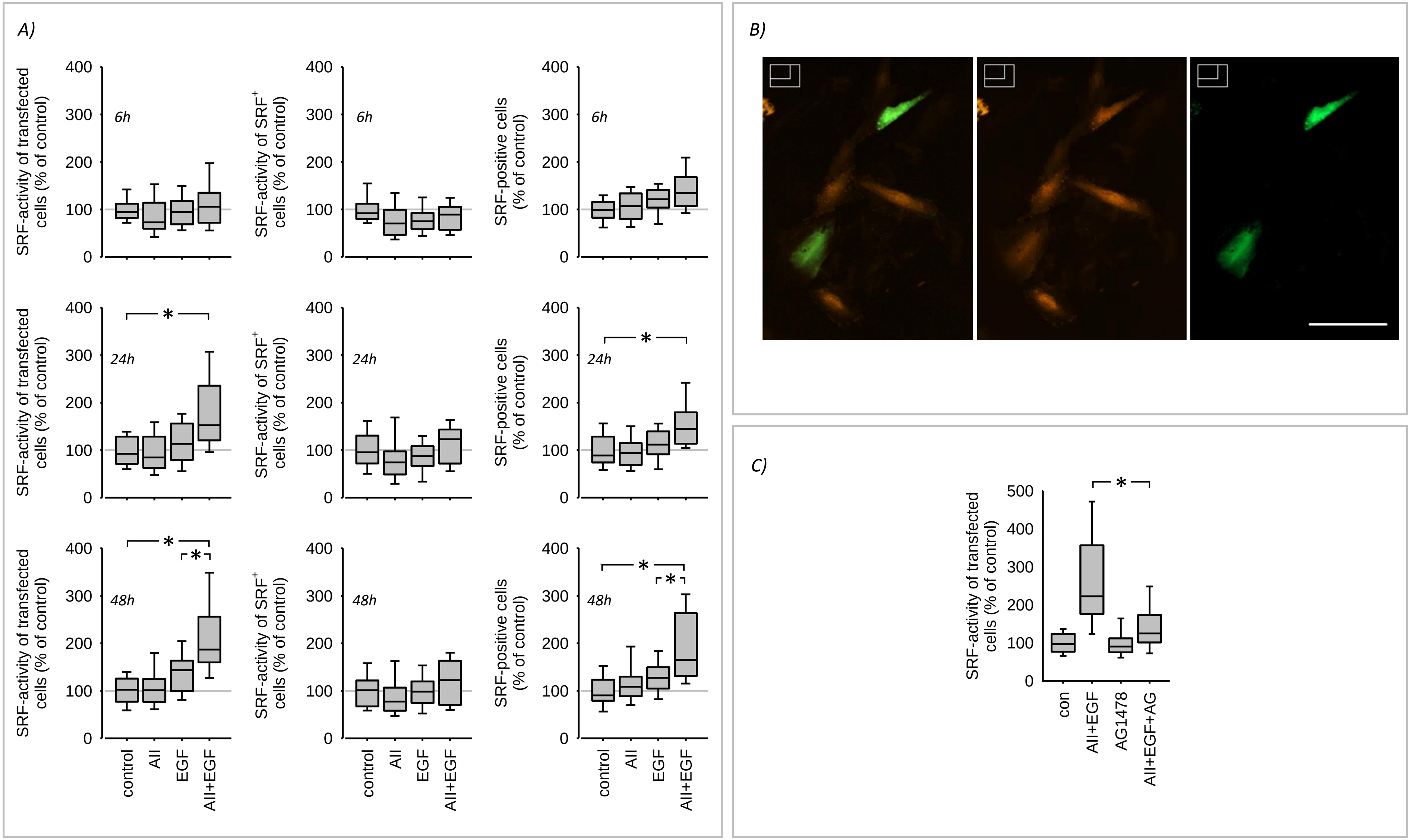

Supplementary Figure SF12. Pharmacology of ATR1-EGFR synergism with respect to SRF activation in HK-2 cells after 6, 24 or 48h. A) Pharmacological screening of putative involved pathway. Inhibition of EGFR-kinase by 100 nmol/l AG1478, inhibition of ERK1/2-phosphorylation by 1 µmol/l U0126, inhibition of cSRC kinase family by 10 µmol/l PP2, inhibition of p38 kinase activity by 1 µmol/l SB203580, inhibition of PI3-kinase activity by 1 µmol/l Ly294002, Ca^{2+}-chelation by 50 µM BAPTA-AM, Rho inhibition with 1 µmol/l Rhosin or 1 µg/ml Rho Inhibitor 1 (RhoI1, cell permeable C3 transferase), inhibition of Rho-kinase (ROCK) activity by 10 µmol/l Y27632 and inhibition of protein kinase C family by 100 nmol/l bisindolylmaleimide (BIM). N=18. * = $p<0.05$ versus AII+EGF. B) Additive inhibitory effects of 100 nmol/l AG1478, 1 µmol/l U0126 and 1 or 10 µmol/l PP2. N=18. * = $p<0.05$ versus AII+EGF. C) Inhibition of actin polymerization by 100 nmol/l latrunculin B (LatB). N=12.

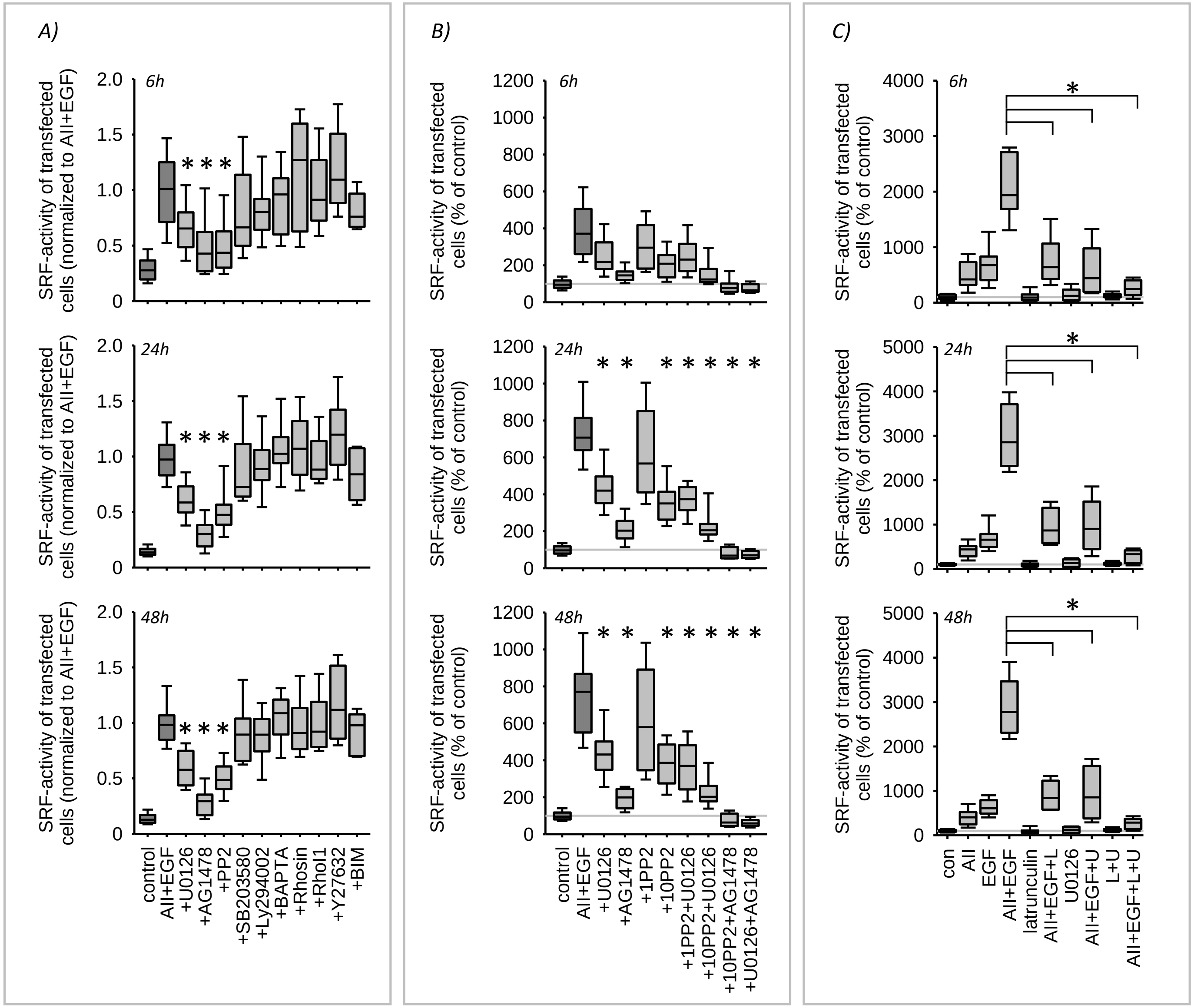

Supplementary figure SF13. A-F) Effect of EGF, AII or AII+EGF on initial EGFR-trafficking in HEK-cells. 300 cells from 3 different passages were measured. G-H) Protein-protein-interaction of AT1R with EGFR determined after 60 minutes by proximity labeling in HEK cells. G) Quantitative evaluation of AT1R-EGFR-interaction. H) Immunoblot examples. 1 nmol/l AII, 10 µg/l EGF. N=12-16. * = p<0.05.

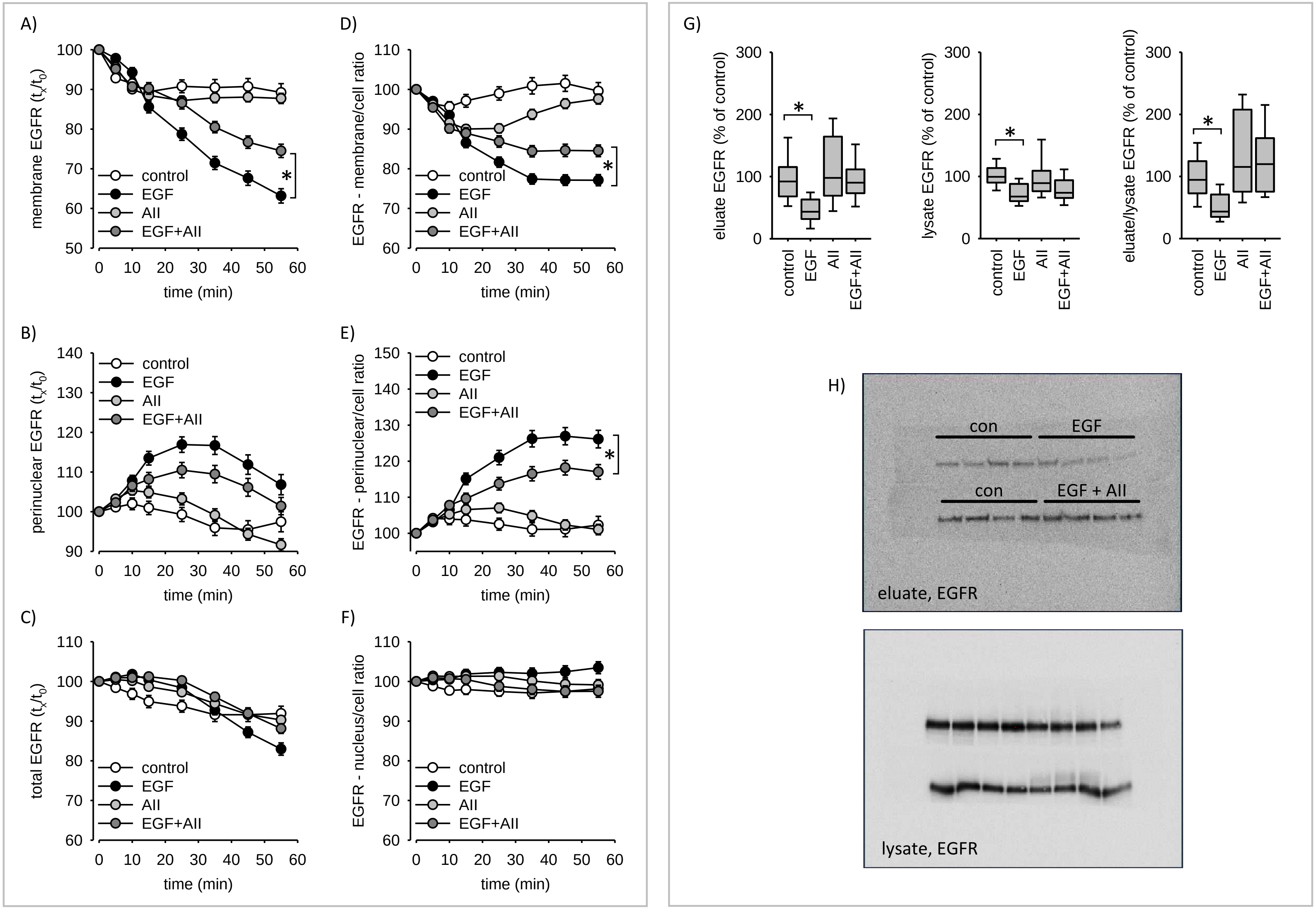

Supplementary figure SF14. Characterization of the two HEK-cell B6 and D5 clones stably expressing AT1R. A) Identification of the two clone stably expressing functional AT1R by SRE reporter assay. t=24h. N=18. * = p<0.05 versus control. B) Synergistic action of AII and EGF on SRF-activity in both cell clones. t=24h. N=12. * = p<0.05. C) ERK1/2-phosphorylation in HEK-AT1R-B6 cells. N=3. D) ERK1/2-phosphorylation in HEK-AT1R-D5 cells. N=3. E) EGFR^{Y1068}-phosphorylation in HEK-AT1R-B6 cells. N=3. F) EGFR^{Y1068}-phosphorylation in HEK-AT1R-B6 cells. N=3. t=30min.

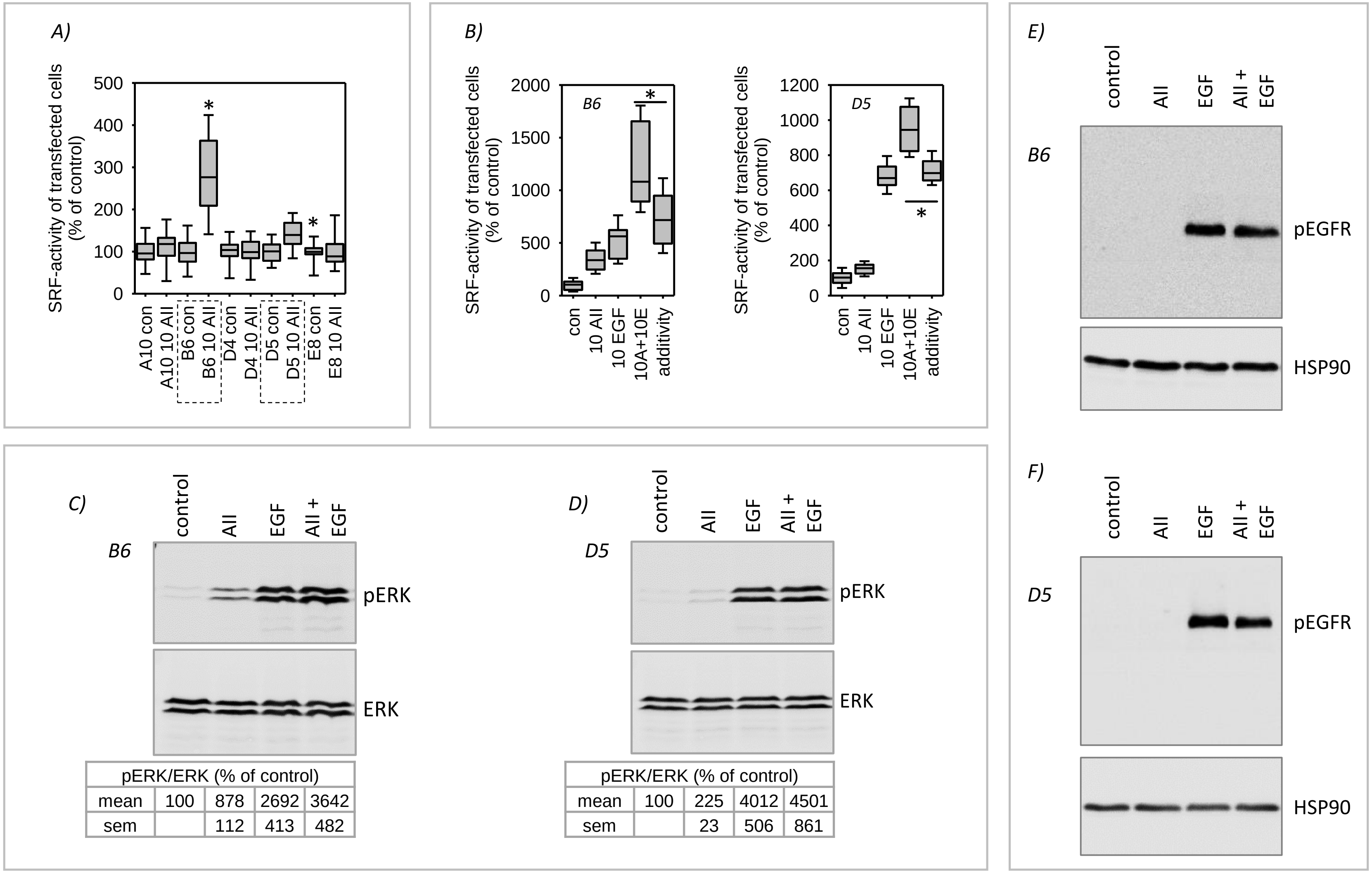

C) B6

pERK/ERK (% of control)				
mean	100	878	2692	3642
sem		112	413	482

D) D5

pERK/ERK (% of control)				
mean	100	225	4012	4501
sem		23	506	861

Supplemetary Figure SF15. Effect of EGF, AII or AII+EGF on the transcriptome in HEK-AT1R-B6 cells, stably transfected with AT1R. Incubarion period = 6h and 24h. N=5 for each condition. A) Number of up- or downregulated RNAs applying the mentioned thresholds. B) RNA-Seq results (fragments per million, FPM) for SRF-induced genes ARC (activity-regulated cytoskeleton-associated protein), EGR1, EGR2, EGR3 (Early growth response protein 1, 2 and 3) and cFOS. t=6h. N=5.

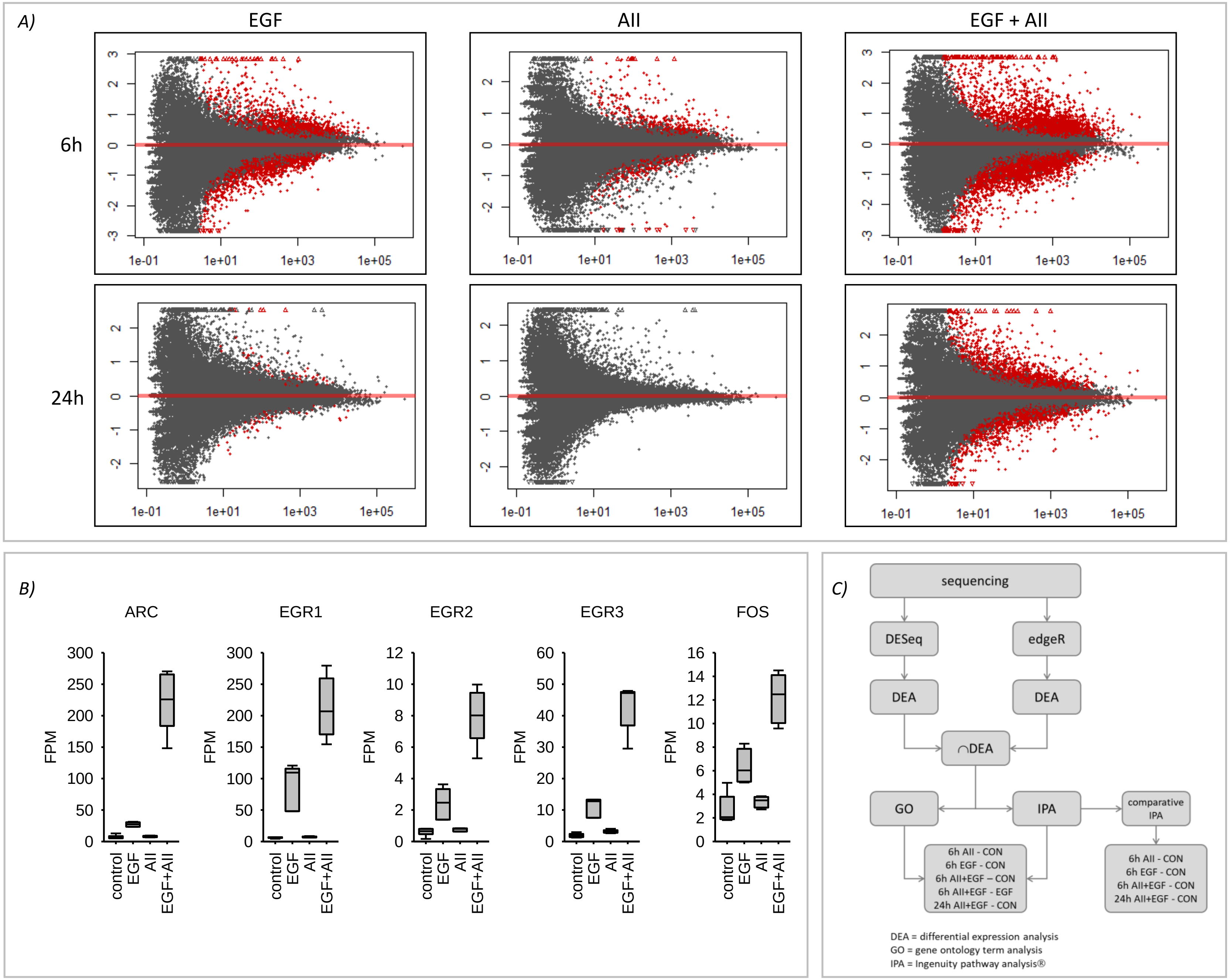

Supplementary Figure SF15. D) Heatmaps showing hierarchical clustering of gene significantly affected by coexposure to EGF and AII after 6h (left panel) or 24h (right panel). Clustering was performed with https://software.broadinstitute.org/morpheus/ using „Eucledian“ metric and „Average“ linkage method.

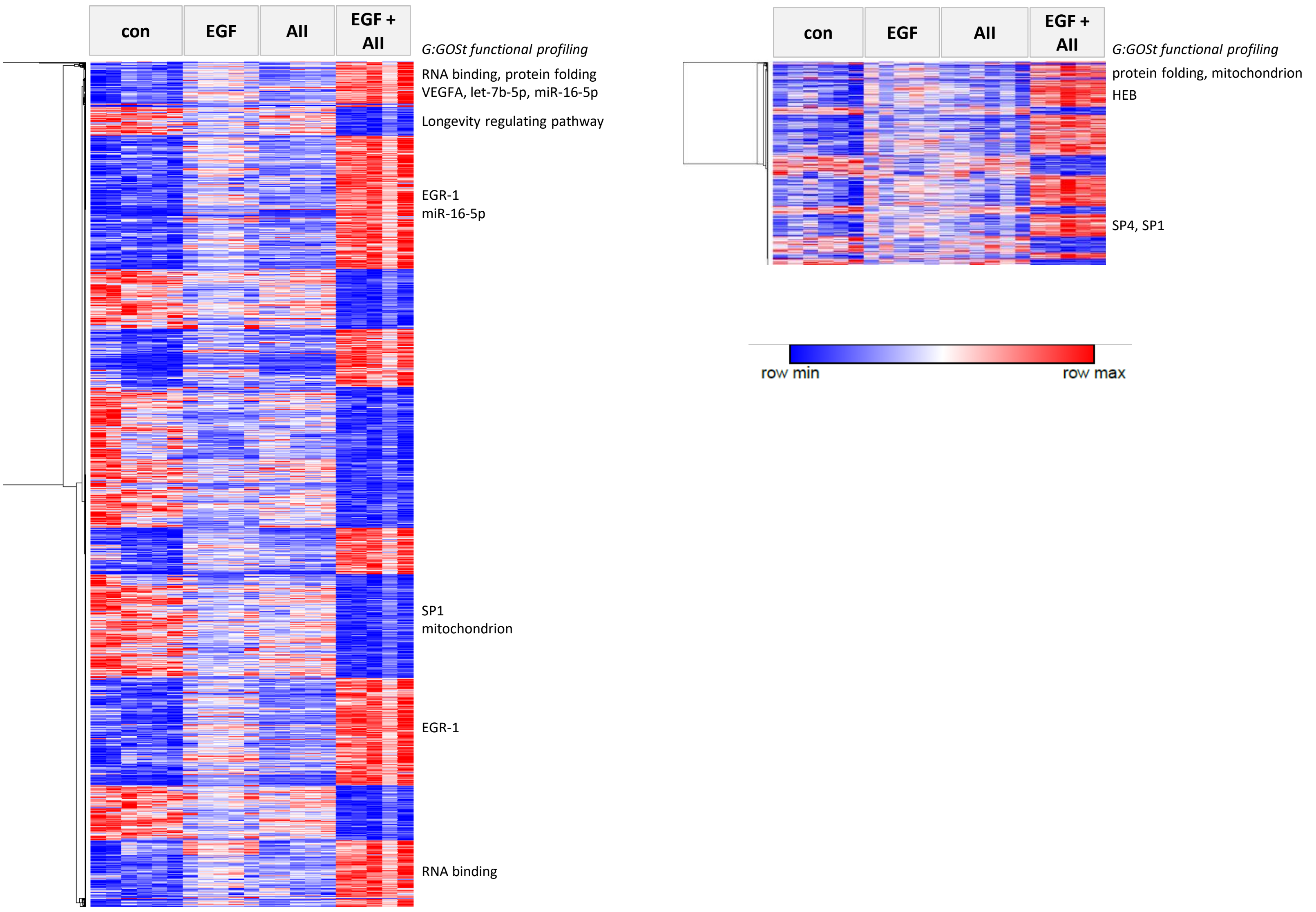

Supplementary Figure SF16. Correlation of gene expression regulation by 10 µg/l EGF or 1 nmol/l with simultaneous AII + EGF incubation after 6h (A) and 24h (B) in HEK-AT1R-B6 cells, stably transfected with AT1R. N=5 for each condition.

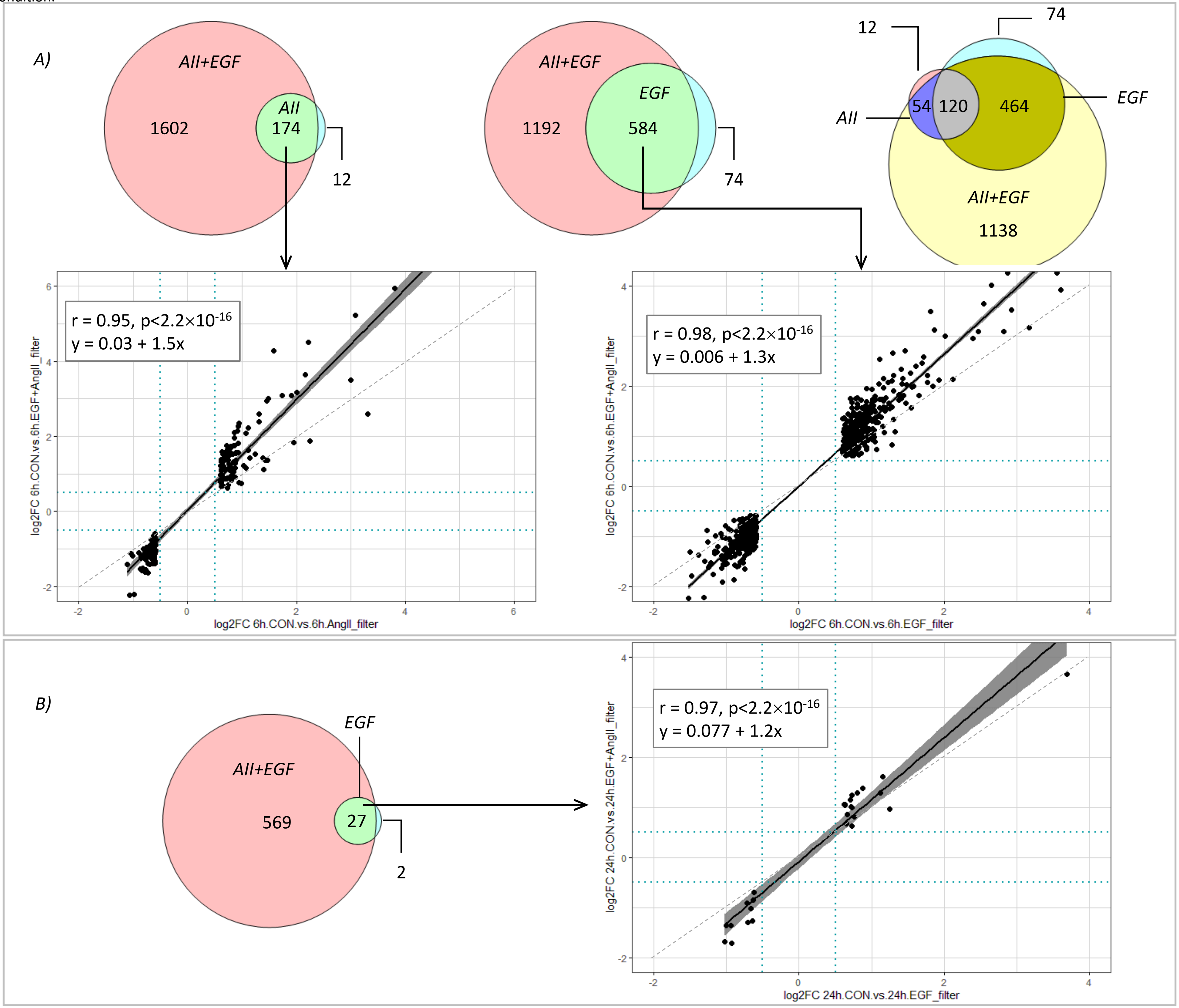

Supplementary Figure SF16. C) Correlation of gene expression regulation after 6h and 24h by 10 µg/l EGF or 1 nmol/l AII + EGF in HEK-AT1R-B6 cells, stably transfected with AT1R. N=5 for each condition.

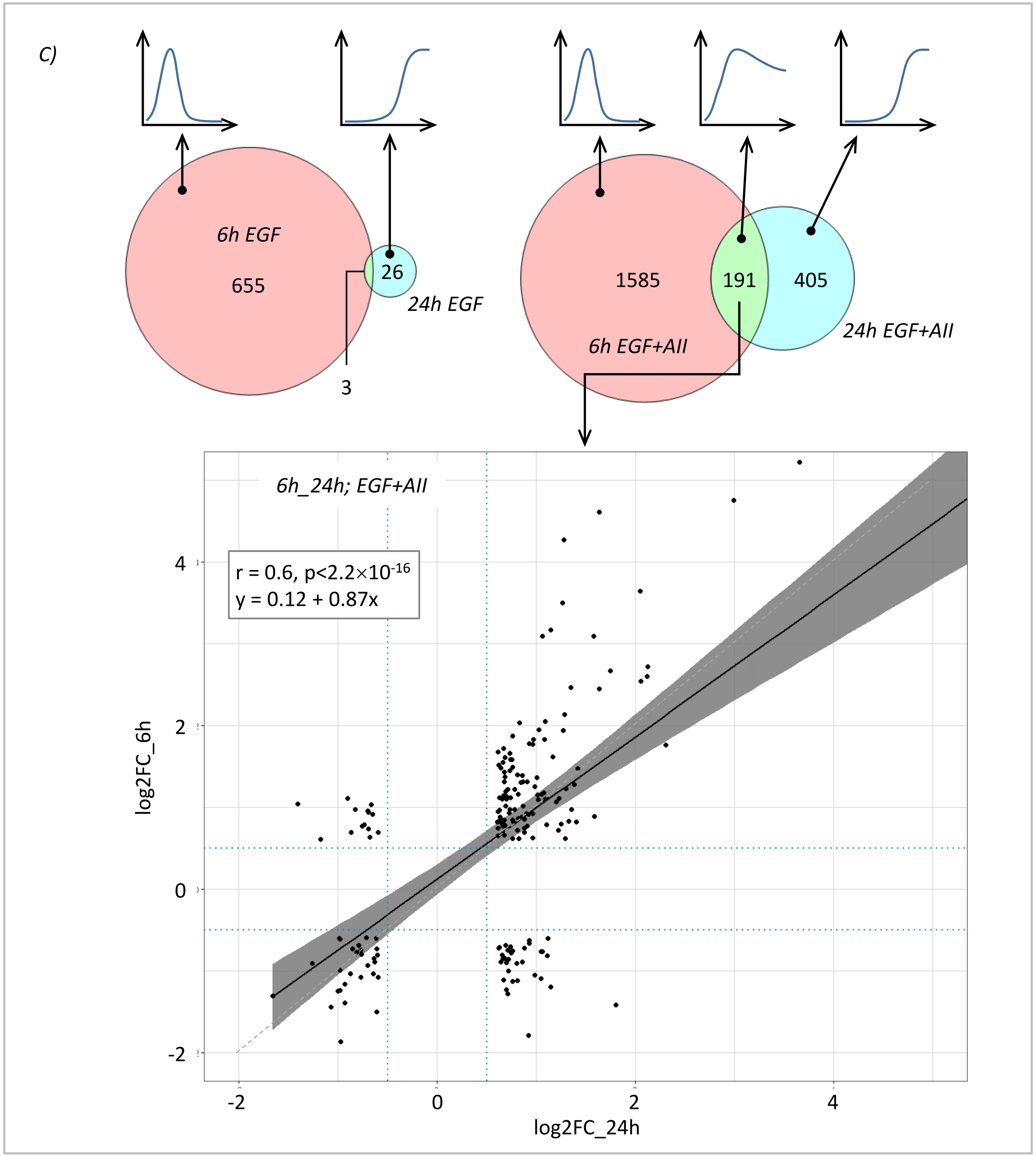

Supplementary Figure SF17. Comparative IPA-analysis of sets of differentially expressed genes in stable transfected HEK293-AT1R clone B6 after simultaneous activation of EGFR and AT1R.

A)

Upstream Analysis | new (6h)

$|Z_{E+A, 6h}| > 2 \cup$ B-$H_{E+A, 6h} < 0.01 \cup$ B-$H_{A, 6h} > 0.01 \cup$ B-$H_{E, 6h} > 0.01$

➥ 72 terms

(B-H_{Top33}: AGT, CCND1, CD3, COMMD1, CSF1, EDN1, F2, F7, FSH, GPER1, Ige, IL15, Lh, MAP2K1/2, MASTL miR-1207-5p, miR-1225-3p, miR-16-5p, miR-296-5p, miR-4640-5p, miR-4755-3p, miR-486-3p, miR-6132, miR-709, miR-7108-3p, NFKB1, NUPR1, Pdgf, PRKCE, TCR, Tgf beta, TGFB3)

Causal networks | new (6h)

$|Z_{E+A, 6h}| > 4 \cup$ B-$H_{E+A, 6h} < 10^{-7} \cup$ B-$H_{A, 6h} > 0.01 \cup$ B-$H_{E, 6h} > 0.01$

➥ 73 terms (50 up, 23 down)

(B-$H_{Top10\text{-}increased}$: CHAD, CIRBP, CXCL5, DTNBP1, HTR2A, IGF2R, KIDINS220, NCOA3, NCOA4, ZMIZ1)

(B-$H_{Top10\text{-}decreased}$: ADAMTS1, CHMP6, MFN2, mir-133, mir-223, PLPP1, SPRED2, STYX, TLE1, VPS25)

Diseases & Function | new (6h)

$|Z_{E+A, 6h}| > 2 \cup$ B-$H_{E+A, 6h} < 0.01 \cup$ B-$H_{A, 6h} > 0.01 \cup$ B-$H_{E, 6h} > 0.01$

➥ 39 terms

(B-H_{Top10}: Cancer, Development of cytoplasm, Fibrogenesis, Formation of brain, Formation of cellular protrusions, Formation of cytoskeleton, Formation of filaments, Lymphatic system tumor, Non-hematological solid tumor, Solid tumor)

Supplementary Figure SF17. Comparative IPA-analysis of sets of differentially expressed genes in stable transfected HEK293-AT1R clone B6 after simultaneous activation of EGFR and AT1R.

B)

Upstream Analysis | new (24h)

$|Z_{E+A,\ 24h}| > 2 \cup \text{B-H}_{E+A,\ 24h} < 0.01$

↪ 39 terms

(incl. 19 miR predicetd to be downregulated; ALDH2, ATF4, CREB1, ERBB2, FGF2, FOS, GPER1, HGF, IFNG, IL1B, IL2, IL5, JUN, MAP2K1, NUPR1, PDGF BB, SP1, TGFB1, Vegf, VEGFA)

Causal networks | new (24h)

$|Z_{E+A,\ 24h}| > 4 \cup \text{B-H}_{E+A,\ 24h} < 10^{-7}$

↪ 19 terms

(increased: ALOX12, CD14, CD151, DLG3, EGFR, G protein, Lpa receptor, MAP3K11, MAP3K8, MUC4, NRG, TAB3, USP5, Vegf, ZP3; decreased: DDIT3, miR-146a-5p, SOSTDC1, TRIM45)

Diseases & Function | new (24h)

$|Z_{E+A,\ 24h}| > 2 \cup \text{B-H}_{E+A,\ 24h} < 0.01$

↪ 51 terms

Causal networks late onset

$|Z_{E+A,\ 24h}| > 4 \cup \text{B-H}_{E+A,\ 24h} < 10^{-7} \cup \text{B-H}_{E+A,\ 6h} > 0.01$

↪ 17 terms

(ALOX12, CD14, CD151, DDIT3, DLG3, G protein, Lpa receptor, MAP3K11, MAP3K8, miR-146a-5p, MUC4, NRG, SOSTDC1, TAB3, TRIM45, USP5, ZP3)

Diseases & Function late onset

$|Z_{E+A,\ 24h}| > 2 \cup \text{B-H}_{E+A,\ 24h} < 0.01 \cup \text{B-H}_{E+A,\ 6h} > 0.01$

↪ 11 terms:

Apoptosis of retinal cells
Development of pancreatic tumor
Endocytosis by eukaryotic cells
Engulfment of cells
Infection by RNA virus
Pancreatic lesion
Pancreatobiliary tumor
Uptake of carbohydrate
Uptake of D-glucose
Uptake of monosaccharide
Viral Infection

Supplementary Figure SF17. Comparative IPA-analysis of sets of differentially expressed genes in stable transfected HEK293-AT1R clone B6 after simultaneous activation of EGFR and AT1R.

C)

Upstream Analysis prolongation

$|Z_{E+A,\ 24h}| > 2 \cup \text{B-H}_{E+A,\ 24h} < 0.01 \cup |Z_{E+A,\ 6h}| > 2 \cup \text{B-H}_{E+A,\ 6h} < 0.01$

➥ 19 terms

(ATF4, CREB1, ERBB2, FGF2, GPER1, HGF, IFNG, IL1B, IL2, IL5, JUN, MAP2K1, miR-185-3p, NUPR1, PDGF BB, SP1, TGFB1, Vegf, VEGFA)

Causal networks prolongation

$|Z_{E+A,\ 24h}| > 4 \cup \text{B-H}_{E+A,\ 24h} < 10^{-7} \cup \text{B-H}_{E+A,\ 6h} < 0.01$

➥ 2 terms (EGFR, VEGF)

Diseases & Function prolongation

$|Z_{E+A,\ 24h}| > 2 \cup \text{B-H}_{E+A,\ 24h} < 0.01 \cup \text{B-H}_{E+A,\ 6h} < 0.01$

➥ 40 terms

Supplementary Figure SF18. Effect of EGFR and AT1R on protein expression of genes involved in EGFR-SRF-siganling in stable transfected HEK293-AT1R clones B6 and D5. Data from 3 independent experiments.

EGFR	protein/ß-actin ratio							
	6h				24h			
	Ctrl	EGF	Ang II	E+A	Ctrl	EGF	Ang II	E+A
**B6**								
V20-010	4,6	0,8	6,0	0,7	9,8	0,1	5,0	0,0
V20-012	48,1	7,8	28,9	2,3	15,5	1,3	16,9	1,6
V20-014	14,4	4,1	29,2	8,1	32,3	3,2	20,1	1,1
**Mean**	**22,4**	**4,2**	**21,4**	**3,7**	**19,2**	**1,6**	**14,0**	**0,9**
**SD**	**10,7**	**1,6**	**6,3**	**1,8**	**5,5**	**0,7**	**3,7**	**0,4**
**D5**								
V20-010	7,0	1,7	7,5	1,4	10,8	0,2	5,2	0,2
V20-012	26,9	7,4	62,8	11,3	54,2	4,1	39,4	2,5
V20-014	6,2	7,2	44,7	6,1	68,5	5,8	35,4	2,9
**Mean**	**13,4**	**5,4**	**38,3**	**6,3**	**44,5**	**3,4**	**26,7**	**1,8**
**SD**	**5,5**	**1,5**	**13,3**	**2,3**	**14,2**	**1,3**	**8,8**	**0,7**

myocardin	protein/ß-actin ratio							
	6h				24h			
	Ctrl	EGF	Ang II	E+A	Ctrl	EGF	Ang II	E+A
**B6**								
V20-010	8,4	6,2	3,7	4,5	1,9	1,1	0,7	0,4
V20-012	23,7	22,3	22,1	18,8	23,0	16,1	16,2	18,0
V20-014	33,3	20,7	23,6	17,8	14,0	18,7	18,0	12,3
**Mean**	**21,8**	**16,4**	**16,5**	**13,7**	**13,0**	**12,0**	**11,6**	**10,2**
**SD**	**5,9**	**4,2**	**5,2**	**3,8**	**5,0**	**4,5**	**4,5**	**4,2**
**D5**								
V20-010	11,7	5,9	6,2	7,5	7,0	6,9	4,3	13,8
V20-012	17,2	19,3	19,9	17,6	14,4	8,4	6,2	8,2
V20-014	21,6	13,8	14,5	11,9	8,7	9,2	11,7	11,8
**Mean**	**16,8**	**13,0**	**13,5**	**12,3**	**10,0**	**8,2**	**7,4**	**11,3**
**SD**	**2,4**	**3,2**	**3,2**	**2,4**	**1,8**	**0,6**	**1,8**	**1,3**

Elk-1	protein/ß-actin ratio							
	6h				24h			
	Ctrl	EGF	Ang II	E+A	Ctrl	EGF	Ang II	E+A
**B6**								
V20-010	0,1	0,1	0,0	0,1	0,1	0,1	0,1	0,0
V20-012	1,0	0,9	0,9	1,0	1,0	1,0	0,8	0,8
V20-014	0,6	0,5	0,8	1,0	0,7	0,6	0,5	0,5
**Mean**	**0,5**	**0,5**	**0,6**	**0,7**	**0,6**	**0,6**	**0,5**	**0,5**
**SD**	**0,2**	**0,2**	**0,2**	**0,3**	**0,2**	**0,2**	**0,2**	**0,2**
**D5**								
V20-010	0,0	0,0	0,0	0,1	0,0	0,0	0,0	0,0
V20-012	0,9	1,0	1,0	1,3	0,6	0,6	0,8	0,8
V20-014	0,5	0,5	0,6	0,8	0,4	0,4	0,4	0,5
**Mean**	**0,5**	**0,5**	**0,6**	**0,7**	**0,3**	**0,4**	**0,4**	**0,4**
**SD**	**0,2**	**0,2**	**0,2**	**0,3**	**0,1**	**0,1**	**0,2**	**0,2**

MRTF-A	protein/ß-actin ratio							
	6h				24h			
	Ctrl	EGF	Ang II	E+A	Ctrl	EGF	Ang II	E+A
**B6**								
V20-010	3,3	8,2	7,5	8,4	6,2	5,1	4,7	1,4
V20-012	10,8	20,1	27,7	26,0	27,7	7,4	5,1	10,5
V20-014	17,2	17,8	15,2	17,0	15,2	16,8	15,0	10,2
**Mean**	**10,4**	**15,4**	**16,8**	**17,1**	**16,4**	**9,7**	**8,3**	**7,4**
**SD**	**3,3**	**3,0**	**4,8**	**4,1**	**5,1**	**2,9**	**2,8**	**2,4**
**D5**								
V20-010	3,2	4,9	5,0	2,7	6,1	5,0	3,9	2,6
V20-012	14,3	13,5	10,1	14,7	16,5	11,1	13,6	8,8
V20-014	8,1	7,3	11,6	12,2	15,9	10,4	6,9	5,1
**Mean**	**8,5**	**8,6**	**8,9**	**9,9**	**12,8**	**8,8**	**8,1**	**5,5**
**SD**	**2,6**	**2,1**	**1,6**	**3,0**	**2,8**	**1,6**	**2,3**	**1,5**

pElk-1	pElk-1/Elk-1							
	6h				24h			
	Ctrl	EGF	Ang II	E+A	Ctrl	EGF	Ang II	E+A
**B6**								
V20-010	0,7	0,7	1,1	0,7	0,8	0,7	0,6	0,5
V20-012	0,3	0,2	0,4	0,2	0,4	0,4	0,4	0,3
V20-014	0,3	0,3	0,3	0,3	0,5	0,4	0,4	0,3
**Mean**	**0,4**	**0,4**	**0,6**	**0,4**	**0,6**	**0,5**	**0,5**	**0,3**
**SD**	**0,1**	**0,1**	**0,2**	**0,1**	**0,1**	**0,1**	**0,1**	**0,1**
**D5**								
V20-010	0,9	0,7	0,8	0,7	1,0	0,9	0,9	0,7
V20-012	0,2	0,2	0,2	0,2	0,3	0,2	0,2	0,4
V20-014	0,3	0,4	0,4	0,4	0,9	1,1	1,2	1,7
**Mean**	**0,5**	**0,4**	**0,4**	**0,4**	**0,7**	**0,7**	**0,8**	**0,9**
**SD**	**0,2**	**0,1**	**0,1**	**0,1**	**0,2**	**0,2**	**0,2**	**0,3**

MRTF-B	protein/ß-actin ratio							
	6h				24h			
	Ctrl	EGF	Ang II	E+A	Ctrl	EGF	Ang II	E+A
**B6**								
V20-010	5,1	4,8	8,1	11,5	10,6	9,6	4,7	2,0
V20-012	17,6	15,1	24,0	31,6	28,2	20,5	15,0	6,1
V20-014	17,1	23,7	32,1	35,4	32,7	35,8	28,6	13,1
**Mean**	**13,3**	**14,5**	**21,4**	**26,2**	**23,9**	**21,9**	**16,1**	**7,1**
**SD**	**3,3**	**4,5**	**5,7**	**6,0**	**5,5**	**6,2**	**5,6**	**2,6**
**D5**								
V20-010	11,2	8,2	5,4	8,0	12,2	7,7	4,6	4,7
V20-012	10,2	10,2	11,8	15,5	17,2	10,1	6,5	6,9
V20-014	14,5	15,8	23,0	26,7	14,6	15,6	17,4	8,9
**Mean**	**12,0**	**11,4**	**13,4**	**16,7**	**14,7**	**11,1**	**9,5**	**6,9**
**SD**	**1,1**	**1,9**	**4,2**	**4,4**	**1,2**	**1,9**	**3,3**	**1,0**

Supplementary Figure SF19. Synergistic effect of EGFR and AT1R on (A) AP1- and (B) EGR--mediated transcriptional activity in HEK293 cells transiently transfected with AT1R. 1 nmol/l AII, 10 µg/l EGF, N(AP1) = 12, N(EGR) = 24. * = p<0.05. (C) ARC protein upregulation by EGF (E) + angiotensin II (A) is prevented to a large extend by U0126 (U) +latrunculin B (L). N=4.

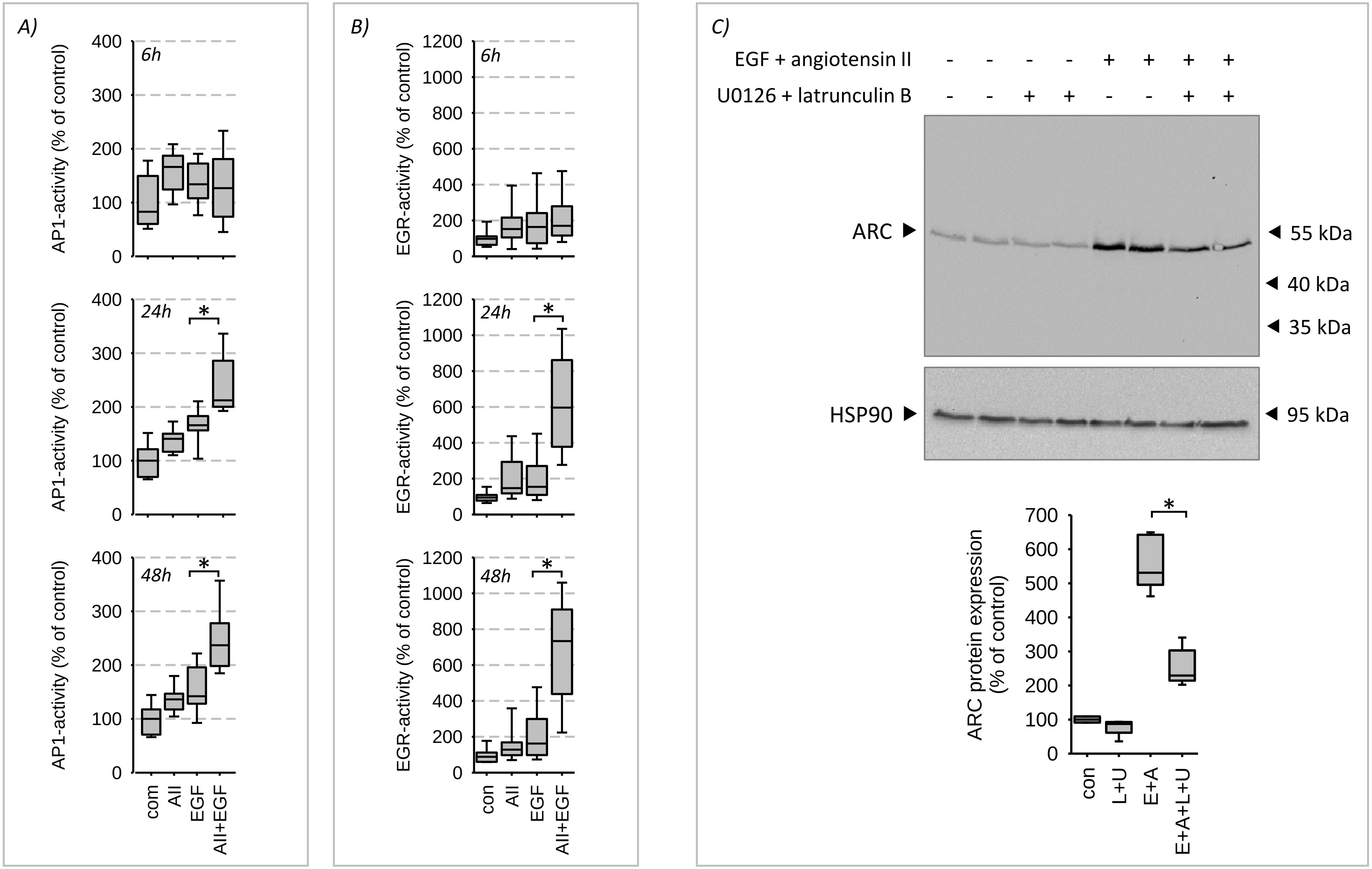

Supplementary Figure SF20. Graphical summary of AT1R-EGFR-synergism and information flow.

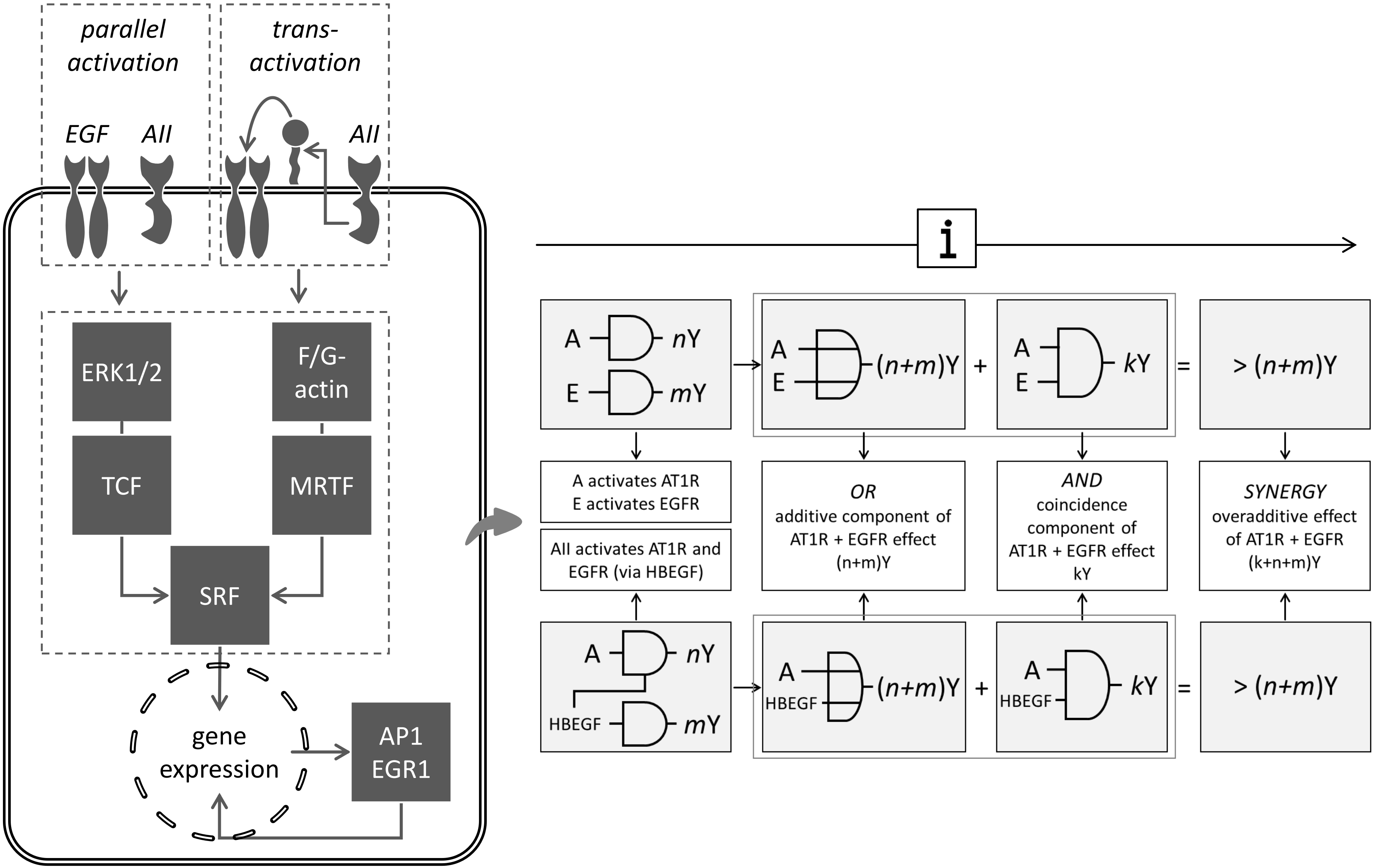

Supplement: Supplementary file 7 — Supplementary file7 (PDF 2531 KB) [file 18_2021_4065_MOESM7_ESM.pdf]
